# CMTM6/PD-L1-targeted AAV promotes anti-tumor immune responses and chemotherapy responsiveness

**DOI:** 10.3389/fimmu.2026.1886034

**Published:** 2026-09-14

**Authors:** Runqiu Chen, Xiaoyu Pang, Fanglin Li, Shanna Wu, Yiting Wang, Xinhang Zheng, Yaqi Zhao, Fengru Qiu, Liying Chen, Fengyu Du, Chen Chen, Rong Zhang, Zhiying Li, Yiru Long, Likun Gong, Meiqing Feng

**Affiliations:** 1 Shanghai Institute of Infectious Diseases and Biosecurity, Fudan University, Shanghai, China; 2 School of Chinese Materia Medica, Nanjing University of Chinese Medicine, Nanjing, Jiangsu, China; 3 State Key Laboratory of Drug Research, Shanghai Institute of Materia Medica, Chinese Academy of Sciences, Shanghai, China; 4 Innovation Center for AI and Drug Discovery, School of Pharmacy, East China Normal University, Shanghai, China; 5 University of Chinese Academy of Sciences, Beijing, China; 6 Zhongshan Institute for Drug Discovery, Shanghai Institute of Materia Medica, Chinese Academy of Sciences, Zhongshan, China; 7 Department of Biological Medicines, School of Pharmaceutical Sciences, Shanghai Engineering Research Center of Immunotherapeutics, Fudan University, Shanghai, China

**Keywords:** adeno-associated virus, chemotherapy, CMTM6, immunotherapy, PD-L1

## Abstract

Chemotherapy is one of the important clinical treatments for malignant tumors, but primary or acquired chemotherapy resistance significantly limits patient benefits. Programmed death ligand 1 (PD-L1) has been reported to be regulated by chemotherapy and to contribute to chemotherapy resistance. However, as a novel regulatory molecule of PD-L1, the relationship between CKLF-like MARVEL transmembrane domain-containing protein 6 (CMTM6) and chemotherapy resistance remains understudied. Here, we found that tumor CMTM6 and PD-L1 were upregulated by chemotherapy in clinical cancer patients, *in vitro* human or mouse tumor models, and *in vivo* mouse tumor models. PI3K-mTOR was the potential mechanism underlying this effect. CMTM6 deficiency could still reduce chemotherapy-induced tumor PD-L1 expression. And CMTM6 could promote tumor cell resistance to chemotherapy-induced apoptosis and growth arrest, which may be associated with p53. *In vivo*, both tumor and host CMTM6 and PD-L1 could promote resistance to chemotherapy, and tumor and host CMTM6 knockout could promoted antitumor immune responses during chemotherapy. Moreover, we developed and evaluated the antitumor effects of CMTM6&PD-L1-targeting adeno-associated virus (AAV), which effectively mobilized antitumor immunity and could be combined with chemotherapy. Our findings reveal that both tumor- and host-derived CMTM6 and PD-L1 are regulatory molecules of chemotherapy resistance and can serve as therapeutic targets for combination therapy with chemotherapy.

## Introduction

Despite the constant emergence of new therapies, chemotherapy remains the primary effective treatment for cancer. For chemotherapy-sensitive tumors such as specific germ cell tumors and lymphomas, a cure can be achieved through chemotherapy alone (1). Traditionally, chemotherapeutic agents exert their antitumor effects primarily by disrupting DNA structure, impeding protein synthesis, inhibiting cell division, and interfering with metabolic processes, and so forth (2–4). Notably, in recent years, chemotherapy has also been found to suppress tumors through novel mechanisms such as promoting tumor immune responses or pyroptosis (5, 6).

Chemotherapy has been found to boost tumor immunogenicity through multiple mechanisms, thereby enhancing antitumor immune responses (7, 8). Chemotherapy drugs primarily act as immunogenic cell death (ICD) inducers by triggering damage-associated molecular patterns (DAMPs) of tumor cells, such as adenosine triphosphate (ATP) and high mobility group box 1 (HMGB1), to recruit and activate immune cells (8–10). Research has also found that chemotherapy upregulates the “eat me” signal calreticulin and the antigen-presenting molecule major histocompatibility complex I (MHC I) in tumor cells, thereby enhancing the antitumor activity of phagocytes and killer cells (11, 12). Dead tumor cells can also release tumor antigens to promote the immune cycle (13). Besides, some studies have reported that low-dose chemotherapy drugs can directly enhance the cytotoxic activity of T cells (14, 15).

However, an undeniable fact is that, primary or acquired chemotherapy resistance have become major factors contributing to poor treatment efficacy and patient prognosis. During chemotherapy, the induction of tumor ICD is accompanied by an upregulation of immune checkpoint molecules such as programmed cell death ligand 1 (PD-L1), which constitutes an important mechanism underlying drug resistance (16). For instance, paclitaxel can induce PD-L1 upregulation via NF-κB signaling, leading to local immune suppression in ovarian cancer and diminishing the antitumor effects of chemotherapy (17). In esophageal squamous cell carcinoma, chemotherapy can increase PD-L1 expression through EGFR/ERK activation, leading to chemotherapy resistance (18). Furthermore, beyond upregulating PD-L1 in tumor cells, chemotherapeutic agents also induce PD-L1 upregulation in immunosuppressive monocytes, thereby inhibiting CD4^+^ T cell activation and diminishing the efficacy of chemotherapy (19). Consequently, novel immunotherapy combination strategies aimed at enhancing chemotherapy sensitivity have garnered significant attention. Extensive clinical data demonstrate that immunotherapy targeting PD-L1 combined with chemotherapy offers patients significantly improved survival outcomes. For instance, the combination of sugemalimab with the chemotherapy drugs capecitabine and oxaliplatin has been shown to substantially extend overall survival by more than three months in patients with advanced gastric cancer (20). Furthermore, multiple immunotherapy-chemotherapy combinations have advanced to Phase III clinical trials for tumors such as squamous non-small cell lung cancer (21–23).

CKLF-like MARVEL transmembrane domain-containing protein 6 (CMTM6), which is an M-type transmembrane protein, is identified as a novel post-translational modification regulator of PD-L1 (24). Research indicates that CMTM6 binds to PD-L1 on the plasma membrane, thereby inhibiting lysosomal degradation and STUB1-mediated ubiquitination degradation of PD-L1 (25, 26). Existing studies indicate that CMTM6 is highly expressed in most common tumors, and patients with high CMTM6 expression often exhibited poor prognosis (27). Furthermore, PD-L1 and CMTM6 exhibit a positive correlation in cancers. Studies indicate that patients with concurrent high expression of both CMTM6 and PD-L1 exhibit a higher response rate to anti-PD-L1 immunotherapy (28). Moreover, our previous studies revealed that CMTM6 regulates antitumor immune responses by maintaining bidirectional PD-1/PD-L1 signaling and through the β-catenin/CCL4 pathway independent of PD-L1 signaling (29, 30). Recent findings also indicate that tumor-derived CMTM6 can also serve as a stable protein for molecules such as CD58 and HER2, and subsequently affect tumor progression (31, 32). These studies further highlight the crucial role of CMTM6 in regulating tumor immunity and tumor therapy, warranting further investigation.

In this study, we aim to systematically investigate the effects of chemotherapy drugs on CMMT6 and PD-L1 expression, and separately explore the relationship between tumor and host CMMT6 and PD-L1 expression and chemotherapy resistance, then to develop gene therapy strategies based on CMTM6/PD-L1 for combination with chemotherapy to achieve enhanced antitumor activity.

## Materials and methods

### Animals

Female BALB/c and C57BL/6 mice aged 6–8 weeks used in animal experiments were purchased from Vital River Laboratory. CMTM6 KO and PD-L1 KO mice were purchased from GemPharmatech and housed in a specific pathogen-free (SPF) environment at the Shanghai Institute of Materia Medica, Chinese Academy of Sciences. All experimental mice were euthanized with carbon dioxide; the euthanasia procedure was performed by a trained veterinarian. The CO_2_ delivery rate was standardized at 40% of the chamber volume per minute. The chamber was not pre-charged with CO_2_; mice were placed in the chamber under atmospheric air, followed by gradual CO_2_ displacement at the regulated volumetric flow rate. Loss of the righting reflex and persistent apnea were monitored continuously; CO_2_ ventilation continued for more than 1 minute after respiratory arrest. A secondary physical euthanasia (cervical dislocation) was performed after CO_2_ exposure to ensure irreversible death, in compliance with animal welfare standards. All experiments were conducted in accordance with the ARRIVE guidelines. Animal care and experiments were conducted according to protocols approved by the Institutional Animal Care and Use Committee (IACUC), approval number 2024-03-GLK-55.

### Cells

RKO, CT26, and 4T1 cells used in the experiment were purchased from Procell; MC38 cells were purchased from BNCC; and HEK293T, B16F10, and A375 cells were purchased from ATCC. RKO cells were cultured in Minimum Essential Medium (MEM; MA0217, Meilunbio) supplemented with 10% fetal bovine serum (FBS; 10100147, Life Technologies) and 1% penicillin/streptomycin (P/S; 15140122, Invitrogen). CT26, MC38, and 4T1 cells were cultured in RPMI1640 medium (MA0215, Meilunbio) supplemented with 10% FBS and 1% P/S. HEK293T, B16F10, and A375 were cultured in Dulbecco’s Modified Eagle Medium (DMEM; MA0212, Meilunbio) supplemented with 10% FBS and 1% P/S. All stable cell lines were constructed using a lentiviral system and maintained under the same conditions as their parental cells. All cells were cultured at 37 °C with 5% CO_2_ in appropriate cell culture incubators and tested for mycoplasma contamination.

### Construction of stable cell lines

All stable cell lines were established using a lentiviral system. A mixture of 7 μg of the target plasmid, 4 μg of the packaging plasmid pMD2.G (VT1443, Youbio), and 4 μg and 3 μg of psPAX2 (VT1444, Youbio) was transfected into HEK293T cells using Lipofectamine 3000 (L3000150; Thermo) to transfect HEK293T cells. Three days later, viruses were harvested using a lentivirus enrichment kit (EMB810A-1; Excellbio) and used to infect tumor cells. Stable cell lines were obtained following selection for puromycin (MA0318; Meilunbio) resistance and were identified by flow cytometry. The sgRNA sequences of the target plasmids were as follows: mouse CMTM6, 5′-CCTGGCCGCCTACTTCGTCC-3′; human CMTM6, 5′-CCGGGTCCTCCTCCGTAGTG-3′.

### Construction of scAAV9

The shCMTM6/PD-L1 scAAV plasmid was constructed in our lab. The pscAAV backbone was linearized via AgeI-HF and EcoRI-HF double digestion, gel-purified, and ligated to annealed CMTM6/PD-L1 shRNA oligos, followed by positive clone screening and plasmid extraction. HEK293T cells at about80% confluency were co-transfected with the recombinant plasmid, pHelper, and Rep/Cap plasmids. Cells and supernatants were collected 48 h post-transfection; crude viral lysate was obtained by PEG8000 precipitation and repeated freeze-thaw cycles. Vector Builder Inc. performed large-scale packaging, purification, viral titration, and quality control following its standard protocols. The specific shRNA sequences used for scAAV9 are as follows: mCMTM6: 5’-CCGAAATGATACACAATTCGACTCGAGTCGAATTGTGTATCATTTCGGTTTTTG-3’; mPD-L1: 5’-TGCCTAACAGAAAGCGTGTCTCGAGACACGCTTTCTGTTAGGCATTTTTG-3’.

### RNA isolation and RT-qPCR analysis

Total cellular RNA was extracted using TRIzol and synthesized into cDNA with an RT kit. Quantitative PCR employed a 2× SYBR Green Fast qPCR mix to assess targeted mRNA expression. The specific primer information is as follows: p53: Forward primer 5’-ATTCAGGCCCTCATCCTCCT-3’; Reverse primer 5’-CCATGGCAGTCATCCAGTCT-3’; GAPDH: Forward primer 5’-TCTCCTGCGACTTCAACA-3’; Reverse primer 5’-TGGTCCAGGGTTTCTTACT-3’; Rptor: Forward primer 5’-ATTGCTCTGCGCTGGTTTTG-3’; Reverse primer 5’-TCCAGTTCAGCTCTCCCAGA-3’; PI3K p110α: Forward primer 5’-TGGCCCATACCAAACAGGAAA-3’; Reverse primer 5’-TGGGCAGCAAGAAGTCAACA-3’. All reactions were performed in triplicate to determine the average Ct value for each gene. GAPDH was selected as the internal control, and the relative expression levels of each gene were calculated using the 2^−ΔΔCt^ method. All primers were synthesized by Rui Mian Biological Technology.

### RNA-Seq

Cas9 control and CMTM6 KO RKO cells were routinely cultured and collected during the logarithmic growth phase. Each cell sample was standardized to more than 5×10^6^ cells to guarantee sufficient RNA quality and quantity for sequencing, with three independent biological replicates included in each group. Total RNA extraction, sample quality inspection, transcriptome library construction, and high-throughput RNA sequencing of all RKO cell samples were performed by BGI Genomics in accordance with standard laboratory workflows. After sequencing, the acquired raw RNA-seq data was subjected to systematic quality control and standardized preprocessing for subsequent transcriptomic profiling. Differential gene expression analysis between Cas9 control and CMTM6 KO RKO cells was performed using the DESeq software package. A corrected p-value of 0.05 was applied as the statistical cutoff, and genes with corrected p-values less than 0.05 were identified as significantly differentially expressed genes in this transcriptomic analysis. These data are available via open access in the Zenodo database (https://doi.org/10.5281/zenodo.21819242).

### Clinical data analysis

Data on the correlation between tumor tissues CMTM6 and PD-L1 expression and chemotherapy drug response were obtained from the GEO database, TCGA database, and Array Express database, with project numbers GSE206501, GSE19293, GSE62321, GSE55410, GSE63885, GSE194040, GSE69657, GSE140494, TCGA-CESC, TCGA-LUAD, E-MEXP-3549, and E-MEXP-1692. Data on changes in CMTM6 expression in tumor patients following chemotherapy treatment were sourced from the GEO database, with project numbers GSE21974, GSE15622, GSE146963, GSE55410, GSE18728, and GSE102120.

### *In vitro* cell experiments

RKO and B16F10 cells were adjusted to a concentration of 5 × 10^5^ cells/mL and seeded into 24-well plates. After 12 hours of incubation, the cells were treated with 1 μM doxorubicin (MB1087, Meilunbio), 0.5 μM etoposide (MB1102, Meilunbio), 1 μM methotrexate (MB1156, Meilunbbio), 1 μM 5-fluorouracil (MB1273, Meilunbio), and 2 μM cisplatin (MB1055, Meilunbio), respectively, for 24 hours, 48 hours, and 72 hours. Finally, the cells were harvested for flow cytometry analysis.

In the dose-dependent experiment, RKO and A375 cells were adjusted to 5×10^5^ cells/mL and seeded into 24-well plates. After 12 hours of static culture, RKO cells were incubated with doxorubicin at concentrations of 0.5 μM, 1 μM, and 1.5 μM for 24 hours. A375 cells were incubated with doxorubicin at concentrations of 1 μM, 2 μM, and 3 μM for 24 hours. Following incubation, cells were harvested for flow cytometric analysis.

In the time-dependent assay, RKO cells were adjusted to a concentration of 5×10^5^ cells/mL, seeded into 24-well plates, and incubated for 12 hours. Subsequently, the cells were treated sequentially with 1 μM doxorubicin at time points of 72 hours, 60 hours, 48 hours, 36 hours, 24 hours, and 12 hours before the end of the experiment. The RKO cells were then harvested for flow cytometric analysis.

For inhibitor screening assays, A375 cells, B16F10 cells, and RKO cells were adjusted to a concentration of 5×10^5^ cells/mL and seeded into 24-well plates. After 12 hours of static culture, 1 μM doxorubicin and corresponding inhibitors were added to the cell culture medium: NFAT Inhibitor-1 (10 nM; HY-P1026, MCE), Cucurbitacin I (10 nM; HY-N1405, MCE), Rapamycin (100 nM; HY-10219, MCE), Capivasertib (10 nM; HY-15431, MCE), Copanlisib (10 nM; HY-15346, MCE), BAY 11-7082 (10 μM; HY-13453, MCE), PD98059 (10 μM; HY-12028, MCE), SP600125 (100 nM; HY-12041, MCE), T-5224 (10 μM; HY-12270, MCE), Pyrrolidinedithiocarbamate ammonium (5 μM; HY-18738, MCE), and JSH-23 (5 μM; HY-13982, MCE). After co-incubation for 48 hours, cells were harvested for flow cytometric analysis.

In the siRNA knockdown experiments for Rptor and PI3K, B16F10 cells were adjusted to a concentration of 5×10^5^ cells/mL and seeded into 12-well plates. After 12 hours of culture, siRNA transfection was performed using Lipofectamine RNAiMAX transfection reagent (13778150, Invitrogen). The cells were cultured for 24 hours post-transfection. A portion of the transfected cells was collected to assess siRNA knockdown efficiency, while another portion was cultured in medium containing 1 μM doxorubicin for 24 hours and then harvested for flow cytometry analysis. siRNA produced by Obio Technology, with the following sequence: si-Rptor-1: sense 5’-GGUCCGAUGUGCAGCUGUAUUTT-3’; antisense 5’-AAUACAGCUGCACAUCGGACCTT-3’; si-Rptor-2: sense 5’-CACGAAAGAUGUUUGACAAAGTT-3’; antisense 5’-CUUUGUCAAACAUCUUUCGUGTT-3’; si-Pi3k-1: sense 5’-CGUAGUGGUAGACGGCGAATT-3’; antisense 5’-UUCGCCGUCUACCACUACGTT-3’; si-Pi3k-2: sense 5’-GUACCGAGAGAUCGACAAATT-3’; antisense 5’-UUUGUCGAUCUCUCGGUACTT-3’; si-NC: sense 5’-UUCUCCGAACGUGUCACGUTT-3’; antisense 5’-ACGUGACACGUUCGGAGAATT-3’.

Cas9 control or CMTM6 KO B16F10 and RKO cells were adjusted to 5×10^5^ cells/mL and seeded into 24-well plates. B16F10 cells were incubated with doxorubicin at 1 μM concentrations, while RKO cells were incubated at 2 μM concentrations. After 24 hours of culture in doxorubicin-containing medium, cells were harvested, stained using an apoptosis detection kit (40302ES60, Yeasen), and analyzed by flow cytometry.

The density of Cas9 control or CMTM6 KO MC38 cells was adjusted to 1×10^5^ cells/mL and seeded into 96-well plates. After a 4-hour incubation, cells were cultured in media containing 0.25 μM doxorubicin, respectively. At 72 hours, the Cell Counting Kit-8 (40203ES60, Yeasen) reagent was added to wells, and absorbance at 450 nm was measured using a microplate reader.

In the scAAV9 qualification experiment, B16F10 cell density was adjusted to 8×10^4^ cells/mL and cultured in a 24-well plate for 12 hours. Subsequently, scAAV9 was added to the cell culture medium at a multiplicity of infection (MOI) of 30,000. On day 5 post-scAAV9 addition, cells were examined under a fluorescence microscope to assess EGFP green fluorescent protein expression. Following this, flow cytometry was employed to detect CMTM6 and PD-L1 expression in the cells.

### Mouse tumor xenograft model

All mouse tumor models for xenografts were established by subcutaneous injection of tumor cells into the dorsal surface of the left forelimb. For the B16F10 and MC38 tumor models, 5×10^5^ tumor cells were inoculated per mouse. For the CT26 and 4T1 tumor models, 1×10^6^ tumor cells were inoculated per mouse. In tumor-bearing models using CMTM6 KO transgenic mice or PD-L1 KO transgenic mice, mice bearing tumors with a volume of approximately 100 mm³ were selected and randomly assigned to experimental and control groups. In other mouse tumor models, random grouping must be performed after tumor implantation before proceeding with subsequent experiments. The tumor volume calculation formula is: Tumor Volume = Length × Width × Width/2. Tumor volume measurements were performed every 1 or 2 days. At the experimental endpoint, tumor-bearing mice were euthanized, and subcutaneous tumor tissue was removed, photographed, and weighed. The tumor tissues were then processed for immunophenotyping analysis via flow cytometry. For survival studies in tumor-bearing mice, the criteria for euthanasia were tumor volume exceeding 2000 mm³, tumor length exceeding 20 mm, or significant weight loss. In animal experiments, the tumor-bearing mouse model received doxorubicin and cisplatin at a dose of 2 mg/kg via intraperitoneal injection. Mice underwent a total of three chemotherapy drug administrations, each spaced 1 day apart. For the scAAV9 administration in the mouse model, the route was peritumoral injection. Mice received injections at 1-day intervals for a total of 3 doses. The doses administered per mouse were 1×10^9^ vg, 3×10^9^ vg, and 6×10^9^ vg, respectively.

### Flow cytometry analysis

As for *in vitro* cell experiments, collected cells were incubated with 4% FBS containing species-specific FC blocker at 4 °C for 20 minutes for blocking. Subsequently, cells underwent 20 minutes of surface marker staining at 4 °C. Following this, cells were treated with BD Cytofix/Cytoperm Buffer (554714, BD Biosciences) at 4 °C for 30 minutes for intracellular marker staining. Finally, cells were analyzed by flow cytometry, and data were processed using NovoExpress software.

In the immunophenotyping analysis experiment, mouse tumor tissues were digested with collagenase IV (40510ES60, Yeasen) and hyaluronidase (20426ES60, Yeasen) at 37 °C and 180 rpm for 40 minutes. The digested tissue was then filtered through a 75-μm nylon mesh (7061011, Dakewe) to prepare a single-cell suspension. This suspension was treated with red blood cell lysis buffer (40401ES60, Yeasen) to remove red blood cells. Cells were blocked with 4% FBS containing anti-mouse CD16/CD32 (101320, BioLegend) at 4 °C for 30 minutes, followed by 20 minutes of surface fluorescent labeling with surface marker antibody solution at 4 °C. Subsequently, cells were treated with BD Cytofix/Cytoperm Buffer (554714, BD Biosciences) and subjected to 30 minutes of intracellular fluorescent labeling at 4 °C. Flow cytometric analysis was performed using ACEA NovoCyte (Agilent), with data processed via NovoExpress software. All flow cytometry antibodies used are listed in **Supplementary Table 1**.

### Statistical analysis

Statistical analysis of data in this study was performed using GraphPad Prism 8.0 software (GraphPad, USA). Independent samples t-tests were used for univariate data comparisons between two groups. ANOVA with Tukey’s multiple comparison correction was employed for univariate data analysis among multiple groups and for multivariate data comparisons involving multiple groups. All statistical analyses were two-tailed tests. Results are expressed as mean ± standard error of the sample mean (SEM). P values < 0.05 were considered statistically significant (*p < 0.05, **p < 0.01, ***p < 0.001).

## Results

### The relationship between tumor CMTM6 expression and chemotherapy in malignant tumors

In recent years, tumor PD-L1 has been reported to be upregulated following chemotherapy and is associated with chemotherapy resistance. As a novel regulatory of PD-L1, we speculated that CMTM6 is also associated with chemotherapy. First, through reanalysis of open-source data from the GEO database, we explored the relationship between CMTM6 expression and chemotherapy responses in clinical malignant tumors, such as colorectal cancer, breast cancer, liver cancer, and ovarian cancer. Compared with the control group, the results showed a trend toward elevated CMTM6 expression levels in most malignant tumors that did not respond to chemotherapy. This phenomenon was particularly evident in colorectal cancer treated with a combination of fluorouracil, irinotecan, and leucovorin, as well as in ovarian cancer treated with decitabine. (**Figure 1A**; **Supplementary Figure 1A**). Additionally, we analyzed PD-L1 expression across these datasets and observed no significant expression differences in PD-L1 for the majority of tumor types. Within these datasets, PD-L1 expression trended upward in chemotherapy-nonresponsive hepatocellular carcinoma (HCC) patients, whereas a downward trend was detected in nonresponders with melanoma and breast cancer (**Supplementary Figure 1B**).

**Figure 1:**
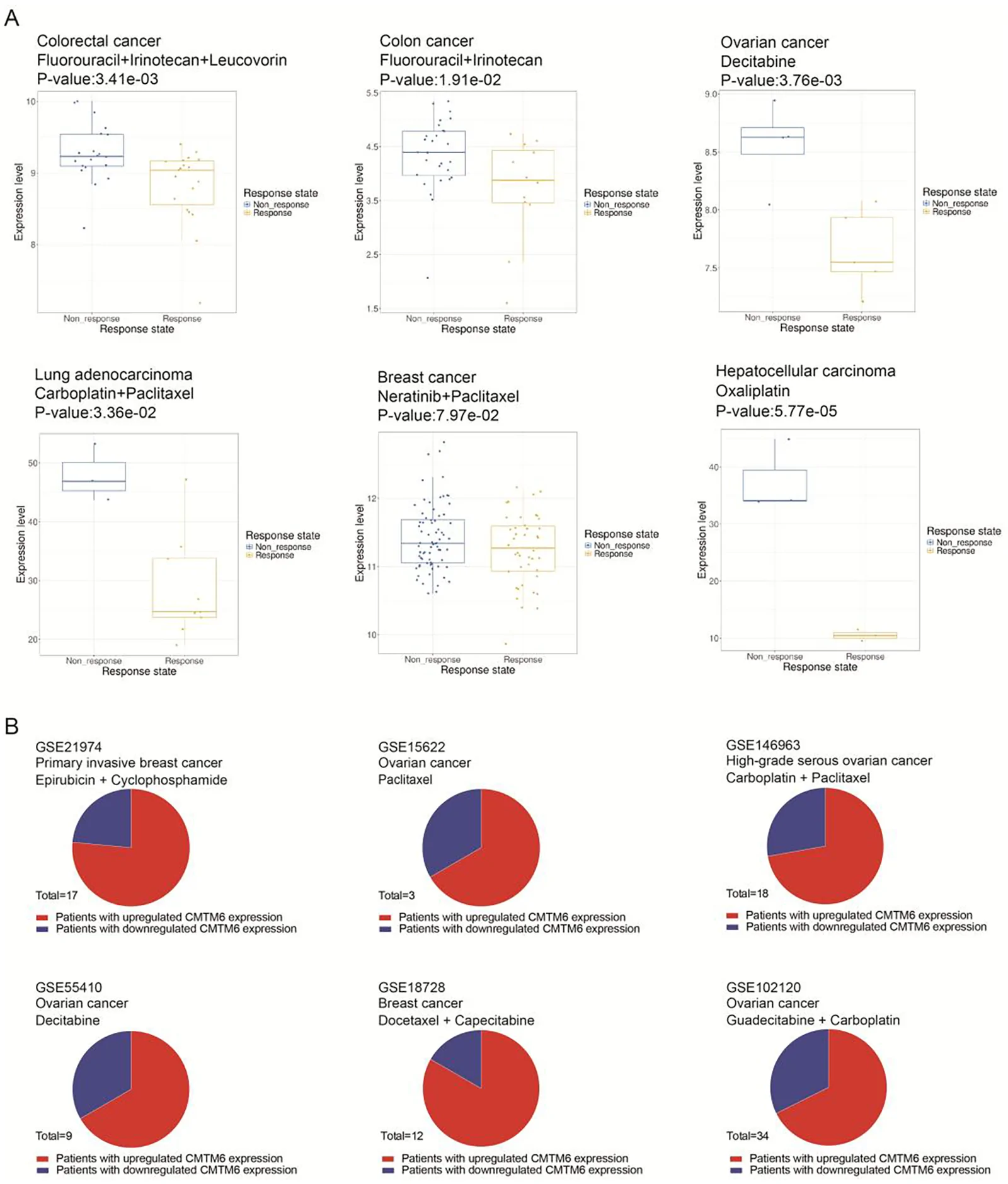
High CMTM6 expression in clinical tumors was negatively correlated with chemotherapy efficacy. **(A)** Correlation between tumor CMTM6 expression and chemotherapy response in multiple tumor types. **(B)** Tumor CMTM6 expression alterations following chemotherapy in patients. The data are presented as the mean ± SEM by unpaired t test.

On the other hand, we also collected open-source GEO datasets containing gene expression data from tumor tissues before and after chemotherapy treatment to investigate the impact of chemotherapy on CMTM6 expression. Compared to the pre-treatment group, the results showed that, in most patients, tumor CMTM6 expression showed an upward trend following administration of chemotherapy drugs, especially in breast cancer and ovarian cancer (**Figure 1B**).

Taken together, these findings suggested that CMTM6 may function as a regulatory molecule for chemotherapy resistance, similar to PD-L1.

### Chemotherapy drugs induced upregulation of CMTM6 expression in tumor cells

To validate findings from clinical data mining, we selected a panel of clinically approved chemotherapy drugs, including doxorubicin (DOX), etoposide (ETO), methotrexate (MTX), fluorouracil (5-FU) and cisplatin (DDP), to observe the effect of chemotherapeutic agents on CMTM6 expression in human and mouse tumor cells. First, we incubated human colorectal cancer cell RKO and mouse melanoma cell B16F10 separately with different chemotherapy drugs for 24 hours. The results showed that CMTM6 expression in these tumor cells was significantly upregulated following treatment (**Figures 2A–C**; **Supplementary Figures 2A, B**), especially in the DOX group. Next, in RKO cells and human melanoma cells A375, DOX can promote CMTM6 expression in a dose-dependent manner (**Figure 2D**).

**Figure 2:**
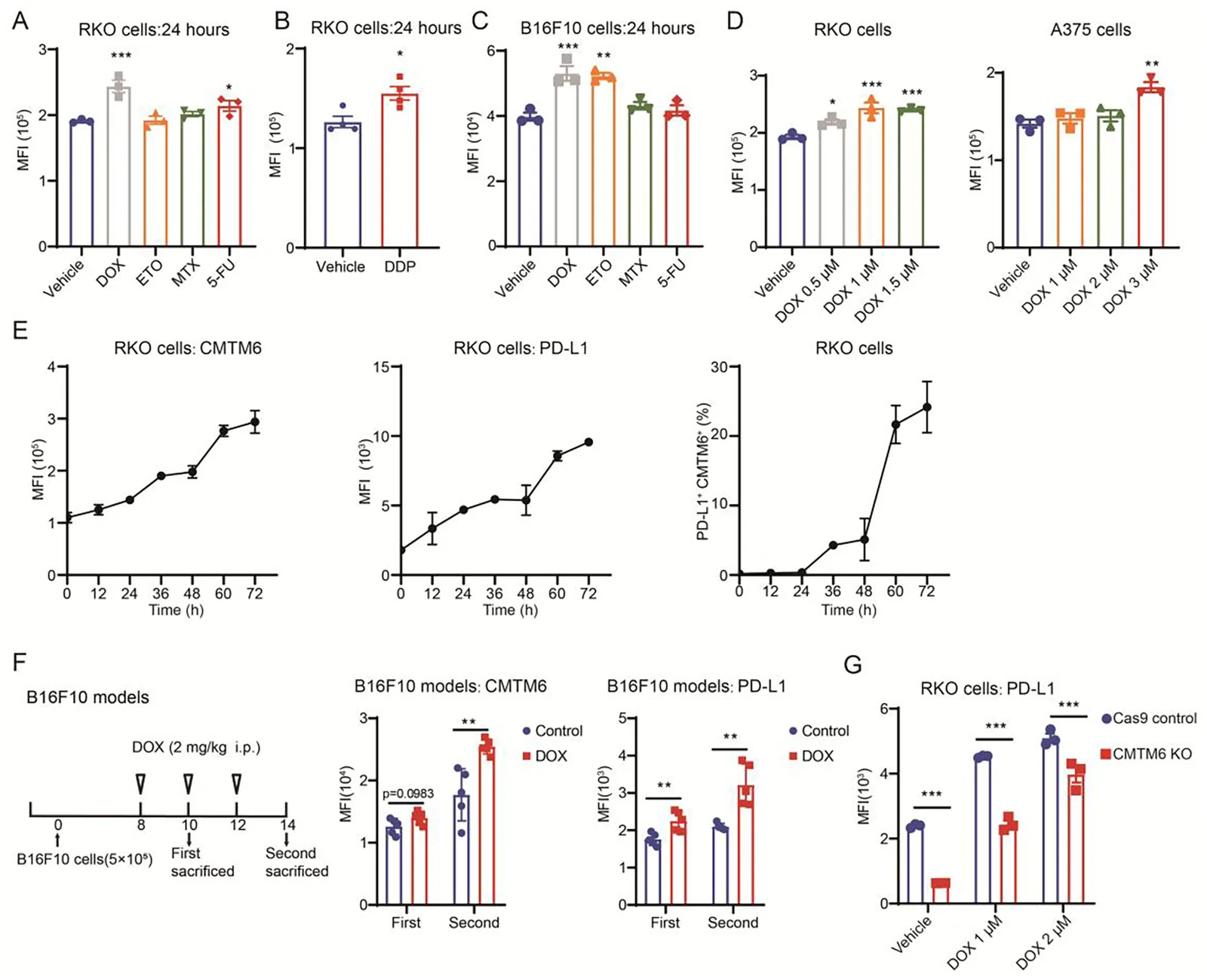
Chemotherapy drugs induced upregulation of CMTM6 and PD-L1 expression in tumor cells. **(A, B, C)** CMTM6 expression in RKO and B16F10 cell models after 24-hour incubation with chemotherapy drugs (A, C: n=3; B: n=4). The control group is the Vehicle group. **(D)** Dose-dependent relationship between CMTM6 expression and chemotherapeutic drugs in RKO and A375 cell models (n=3). The control group is the Vehicle group. **(E)** Time-dependent relationship between CMTM6 and PD-L1 expression in RKO cell models and chemotherapy drug incubation time (n=3). **(F)** CMTM6 and PD-L1 expression in tumors of B16F10 tumor-bearing mice during doxorubicin treatment (n=5). **(G)** PD-L1 expression levels in RKO cells induced by different doses of doxorubicin incubation in Cas9 control and CMTM6 KO cells (n=3). The data are presented as the mean ± SEM. *p < 0.05; **p < 0.01; ***p < 0.001; ns not significant by one-way/two-way ANOVA followed by Tukey’s multiple comparisons test.

Subsequently, we investigated the dynamic changes in CMTM6 expression induced by chemotherapy drugs and its relationship with PD-L1. As DOX treatment time elapsed, CMTM6 levels in RKO cells gradually increased, reaching nearly 3-fold the baseline level at 72 hours (**Figure 2E**). We also found that DOX stimulated PD-L1 expression in RKO cells in a time-dependent way (**Figure 2E**). Interestingly, the proportion of tumor cells with CMTM6 and PD-L1 co-expression induced by DOX also exhibited an upward trend, and the magnitude of this increase was nearly 30% (**Figure 2E**). Of course, there were still many cells that only elevate CMTM6 or only elevate PD-L1.

To explore whether chemotherapeutic agents are also capable of inducing CMTM6 and PD-L1 upregulation in tumor cells *in vivo*, we treated B16F10- and mouse colorectal cancer MC38-tumor-bearing mouse models with DOX and evaluated the expression levels of both molecules in tumor tissues at two distinct time points (**Figure 2F**; **Supplementary Figure 3A**). Flow cytometry results demonstrated that DOX treatment elicited CMTM6 and PD-L1 upregulation in murine tumor cells, with their expression levels rising progressively over the course of chemotherapy (**Figure 2F**; **Supplementary Figure 3A**).

Then, based on the regulatory role of CMTM6 on PD-L1, we sought to determine whether chemotherapy-induced PD-L1 upregulation in tumors is also modulated by CMTM6. We established a CMTM6-knockout (KO) RKO cell line via CRISPR-Cas9 technology (**Supplementary Figure 3B**), and incubated it with 1 μM and 2 μM doxorubicin, separately. In vehicle group, the CMTM6 deficiency effectively reduced PD-L1 membrane expression in RKO cells (**Figure 2G**). After DOX induction, PD-L1 levels remained significantly elevated, and marked differences in PD-L1 expression levels persisted between the Cas9 control and CMTM6 KO groups, indicating that CMTM6 retained its capacity to regulate chemotherapy-elicited PD-L1 expression (**Figure 2G**; **Supplementary Figure 2C**). However, the extent to which CMTM6 deficiency affected PD-L1 membrane expression decreased with increasing DOX treatment concentrations (**Figure 2G**). This may be related to the fact that a sizable proportion of RKO cells exhibited asynchronous upregulation of CMTM6 and PD-L1 following chemotherapy treatment.

Taken together, these observations indicated that chemotherapeutic drugs could trigger the upregulation of both tumor CMTM6 and PD-L1 *in vitro* and *in vivo*, and CMTM6 could still regulate chemotherapy-induced PD-L1 levels.

### Potential mechanisms underlying chemotherapy-induced tumor CMTM6 upregulation

The above results indicate that chemotherapy can induce CMTM6 expression in tumors. Therefore, we further investigated the mechanism by which chemotherapy drugs promote the upregulation of CMTM6 in tumors. Given that chemotherapy drugs not only upregulate CMTM6 expression but also enhance PD-L1 levels, we explored the potential mechanisms underlying chemotherapy-induced CMTM6 upregulation by examining key reported signaling pathways known to upregulate PD-L1, including the ERK, JAK/STAT, NF-κB, and PI3K/Akt pathways. We performed a pathway screen using specific inhibitors targeting key signaling nodes. In the B16F10 model, compared with the doxorubicin group, CMTM6 expression decreased by 21% and 23%, respectively, after the cells were treated with doxorubicin and either the mTOR inhibitor rapamycin or the PI3K inhibitor copanlisib (**Figure 3A**). A comparable regulatory trend was observed in the A375 cells and RKO cells (**Figure 3B**; **Supplementary Figure 3C**). Of course, mTOR inhibitors and PI3K inhibitors also successfully downregulated chemotherapy-induced PD-L1 expression (**Figures 3A, B**). Subsequently, we used siRNA to knockdown Rptor (mTOR pathway) or PI3K p110α (PI3K pathway) in B16F10 tumor cells, followed by co-incubation with doxorubicin. Compared with the control group treated with si-NC combined with doxorubicin, knocking down Rptor or PI3K p110α significantly inhibited the doxorubicin-induced upregulation of CMTM6 and PD-L1 expression in B16F10 tumor cells (**Supplementary Figures 3D, E**). These findings suggested that the mTOR and PI3K pathways may represent potential mechanisms underlying chemotherapy-induced upregulation of CMTM6.

**Figure 3:**
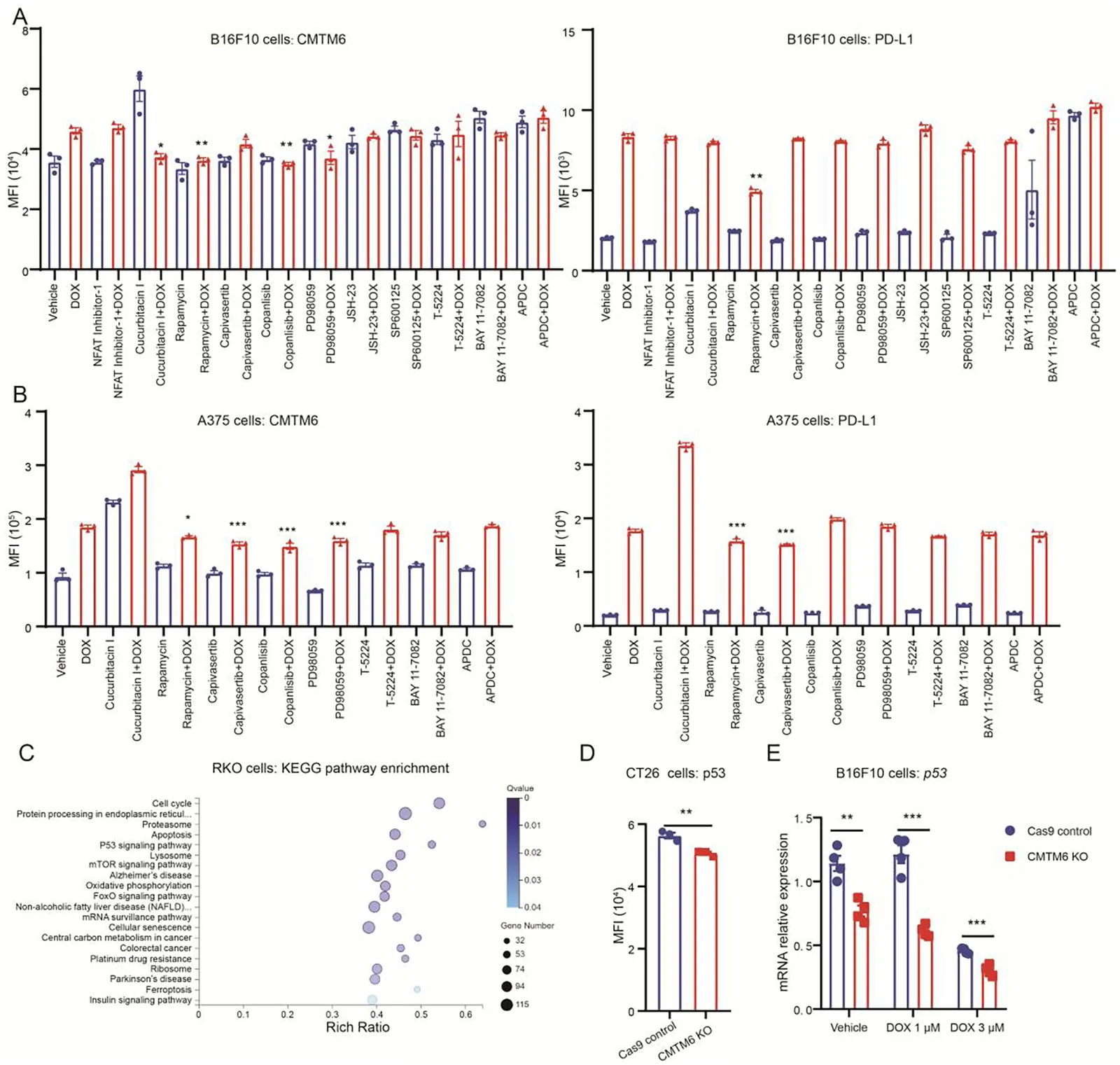
The relationship between CMTM6, PI3K-mTOR, and P53 in chemotherapy treatment. **(A)** Expression of CMTM6 and PD-L1 in B16F10 cells after 48 h treatment with various pathway inhibitors and DOX (n=3). The control group is the DOX group. **(B)** Expression of CMTM6 and PD-L1 in A375 cells after 48 h treatment with various pathway inhibitors and DOX (n=3). The control group is the DOX group. **(C)** KEGG enrichment analysis of the RKO cell line with Cas9 control and CMTM6 KO (n=3). **(D)** Protein p53 expression in CT26 cell lines with Cas9 control and CMTM6 KO (n=3). **(E)** p53 mRNA expression in B16F10 cells under different doxorubicin doses in the Cas9 control and CMTM6 KO groups (n=4). The data are presented as the mean ± SEM. *p < 0.05; **p < 0.01; ***p < 0.001 by unpaired t test or one-way/two-way ANOVA followed by Tukey’s multiple comparisons test.

### Tumor CMTM6 was a potential stabilizer of p53

To further investigate the effects of CMTM6 expression on tumor cells, we performed transcriptome sequencing analysis on the established Cas9 control RKO cells and CMTM6 knockout RKO cells. KEGG pathway enrichment analysis of differentially expressed genes between the two cell populations revealed that significant alterations have occurred in pathways such as the cell cycle, apoptosis, p53 signaling pathway, mTOR signaling pathway, FoxO signaling pathway, cellular senescence and platinum drug resistance (**Figure 3C**). It was noteworthy that these pathways have been frequently reported to be associated with chemotherapy resistance. For example, p53 has been reported to maintain genomic stability in tumor cells, thereby promoting chemotherapy resistance (33, 34). Furthermore, in the mouse colorectal cancer CT26 cell model, via comparison p53 of Cas9 control cells and CMTM6 KO cells by flow cytometry analysis, we found that the loss of tumor CMTM6 affected p53 expression. (**Figure 3D**). Subsequently, in the B16F10 cell model, CMTM6 loss still caused downregulation of tumor p53 RNA transcription levels, and this trend remained significant after doxorubicin incubation (**Figure 3E**).

Taken together, we proposed that CMTM6 deficiency in tumors was associated with multiple mechanisms of chemotherapy resistance, and tumor CMTM6 was a potential stabilizer of p53.

### Tumor CMTM6 regulated chemotherapy-mediated apoptosis and growth inhibition in tumor cells

Given that the pathways presented in the preceding results, including the mTOR pathway, participate in the regulation of apoptosis and chemoresistance, we further investigated whether CMTM6 expression affected the antitumor efficacy of chemotherapeutic agents. First, we compared chemotherapy-induced apoptosis between Cas9 control tumor cells and CMTM6 KO tumor cells by Annexin V-FITC/PI staining. In the RKO cell model, following treatment with 2 μM DOX, CMTM6 deficiency increased tumor cell death from15.6% to 23.6% (**Figures 4A, B**). Similarly, in the B16F10 cell model, treatment with 2 μM DOX resulted in significantly increased tumor cell death in CMTM6-deficient cells (**Figures 4C, D**). Besides, we also compared chemotherapy-induced cell growth arrest between Cas9 control tumor cells and CMTM6 KO tumor cells by cell counting kit-8 (CCK-8) assays. Results showed that in the MC38 cell model, 72 hours after DOX treatment, the growth rate of CMTM6 KO MC38 cells was significantly slower than that of control cells (**Figure 4E**).

**Figure 4:**
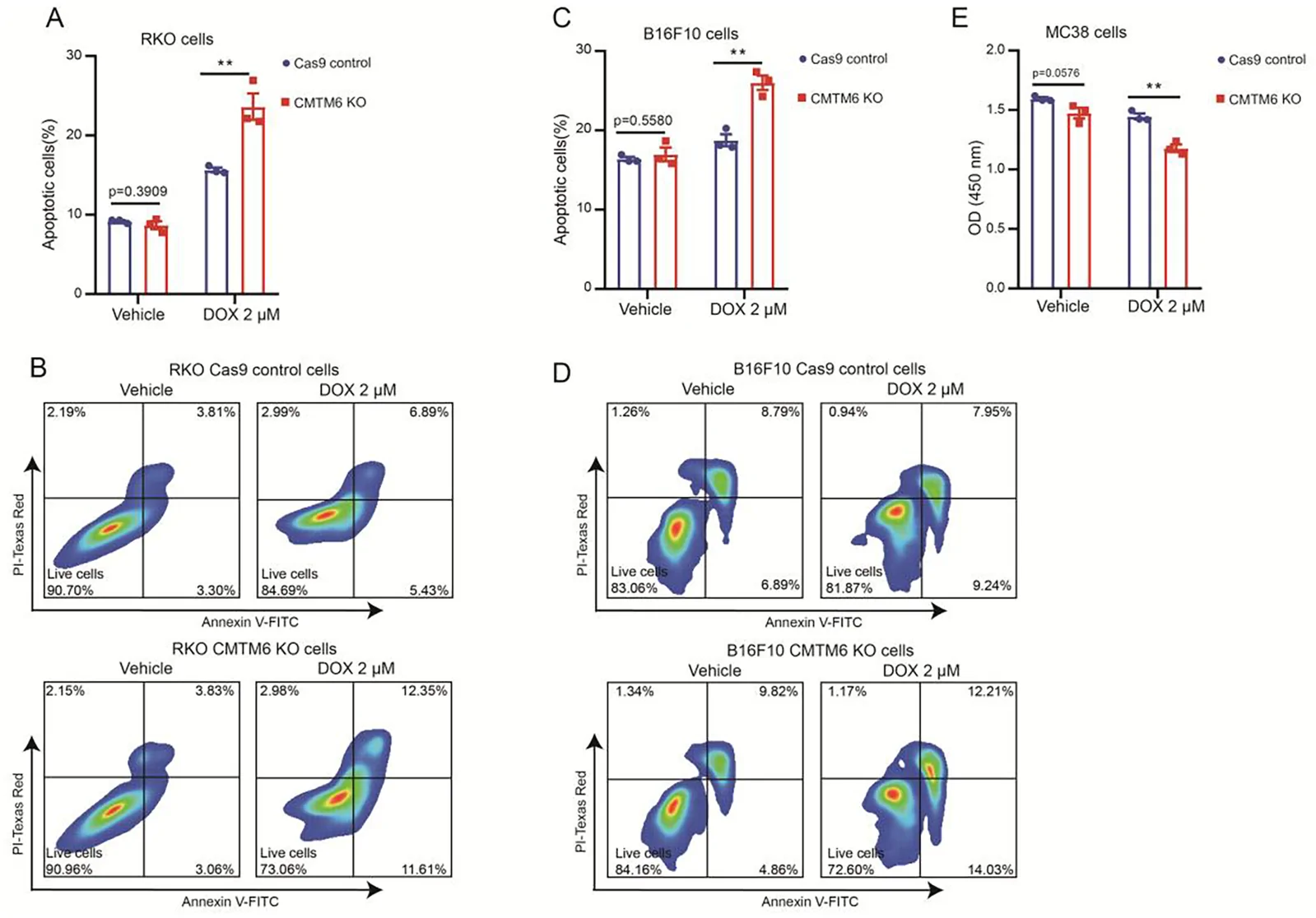
CMTM6 deficiency in tumors enhanced chemotherapy-induced tumor cell apoptosis and growth inhibition. **(A)** Statistical chart for apoptosis in Cas9 control and CMTM6 KO RKO cells under doxorubicin incubation (n=3). **(B)** Representative flow cytometry density plots for apoptosis in Cas9 control and CMTM6 KO RKO cells under doxorubicin incubation. **(C)** Statistical chart for apoptosis in Cas9 control and CMTM6 KO B16F10 cells under doxorubicin incubation (n=3). **(D)** Representative flow cytometry density plots for apoptosis in Cas9 control and CMTM6 KO B16F10 cells under doxorubicin incubation. **(E)** Cell proliferation analysis of Cas9 control and CMTM6 KO MC38 cells after co-incubation with doxorubicin for 72 hours (n=3). The data are presented as the mean ± SEM. **p < 0.01; ***p < 0.001 by two-way ANOVA followed by Tukey’s multiple comparisons test.

Based on these experimental findings, we concluded that tumor CMTM6 could regulate chemotherapy-induced apoptosis and growth suppression in tumor cells.

### Tumor CMTM6 regulated the *in vivo* efficacy of chemotherapy drugs

Considering that CMTM6 correlated with DOX-mediated tumor suppression *in vitro*, to assess the role of tumor CMTM6 in regulating the *in vivo* efficacy of chemotherapeutic agents, we proposed to carry out follow-up experiments using murine tumor cell line-derived xenograft (CDX) models in immunocompetent mice. Of note, our prior findings have confirmed that tumor-specific CMTM6 knockout results in marked suppression of tumor growth *in vivo (*29). To eliminate differences in tumor growth between the Cas9 control group and the CMTM6 KO group, before the doxorubicin administration, we selected mice from the two groups to maintain consistency in the initial progression of the tumor. In the mouse breast cancer 4T1 models, as for the vehicle groups, based on the initial mice selection, we observed no significant differences in subsequent tumor growth between the Cas9 control group and the CMTM6 KO group (**Figure 5A**). Notably, following doxorubicin administration, tumor growth was significantly suppressed in the CMTM6 KO group. The average tumor suppression rate of doxorubicin in the Cas9 control group was 32%, whereas it increased to 53% after CMTM6 KO (**Figure 5A**). We also found that CMTM6 and PD-L1 expression remained upregulated in 4T1 tumors following doxorubicin administration, consistent with our previous findings (**Figure 5B**). In the MC38 tumor-bearing mouse model, a trend consistent with the 4T1 model was observed, in which CMTM6 KO significantly augmented the antitumor efficacy of DOX (**Figure 5C**).

**Figure 5:**
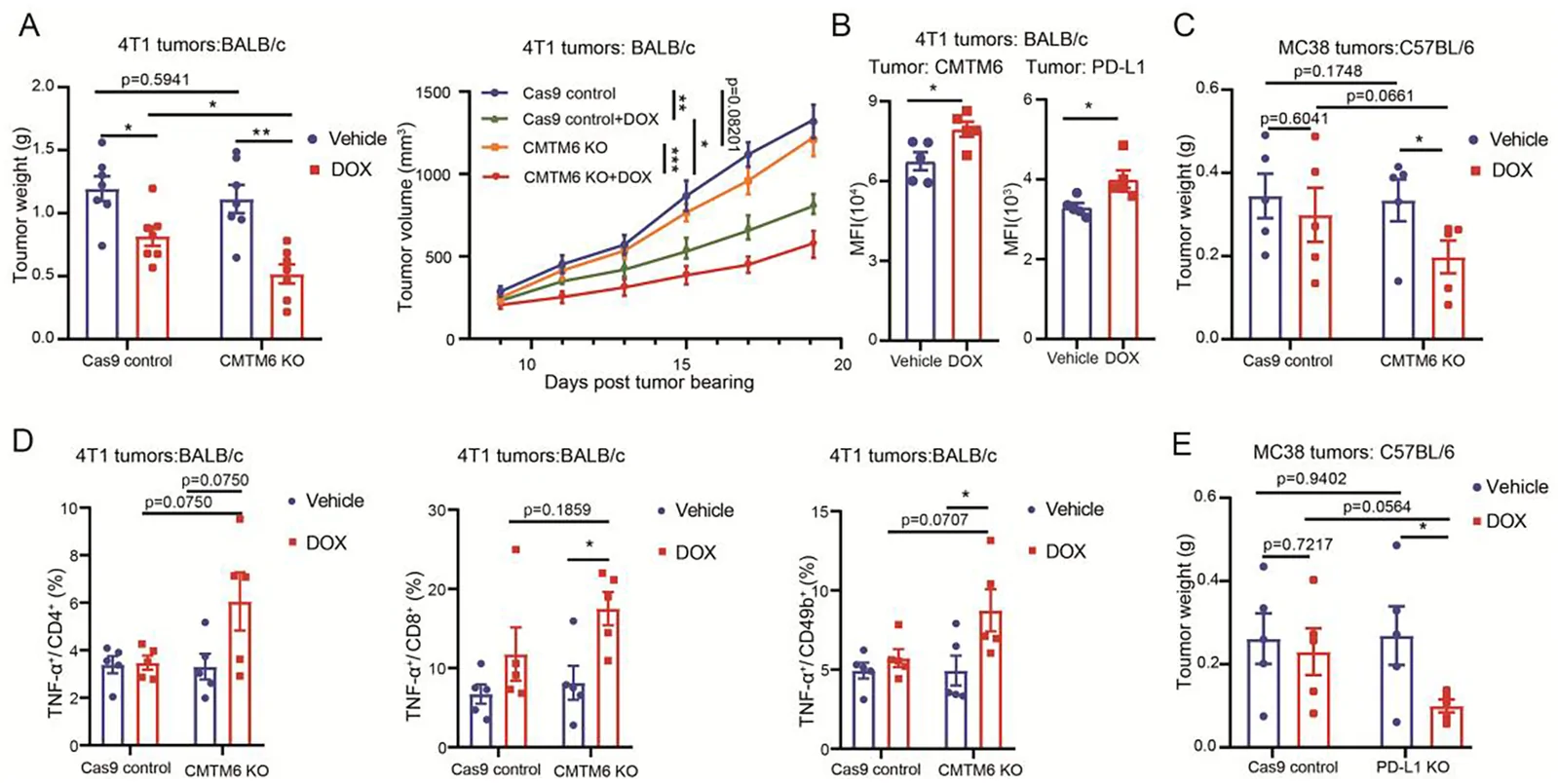
CMTM6 deficiency in tumors enhanced the antitumor efficacy of doxorubicin. **(A)** End-point tumor weight and tumor growth curves in Cas9 control and CMTM6 KO 4T1 tumor-bearing mouse models following doxorubicin treatment (n=7). **(B)** Expression of CMTM6 and PD-L1 in tumors of 4T1-bearing mice treated with doxorubicin (n=5). **(C)** Final tumor weight in MC38 tumor-bearing mice with Cas9 control and CMTM6 knockout following doxorubicin treatment (n=5). **(D)** Cas9 control and CMTM6 KO 4T1 tumor-bearing models. Flow cytometric analysis of TNF-α expression in CD4^+^ T cells, CD8^+^ T cells, and NK cells following doxorubicin treatment (n=5). **(E)** Final tumor weight in Cas9 control and PD-L1 KO MC38 tumor-bearing mice following doxorubicin treatment (n=5). The data are presented as the mean ± SEM. *p < 0.05; **p < 0.01; ***p < 0.001 by one-way ANOVA followed by Tukey’s multiple comparisons test.

Subsequently, we analyzed the lymphocytes function in the 4T1 tumor immune microenvironment following doxorubicin treatment using flow cytometry. The results showed that doxorubicin therapy induced marked upregulation of tumor necrosis factor α (TNF-α) in CD4^+^ T cells, CD8^+^ T cells and NK cells within the CMTM6 KO group (**Figure 5D**). This may be due to CMTM6 deficiency promoting chemotherapy-induced tumor cell death, thereby facilitating tumor antigen release, or to CMTM6 deficiency lifting tumor-associated immunosuppression, as we reported before.

Given that CMTM6 could regulate chemotherapy-induced membranal PD-L1 levels, we also hypothesized that CMTM6 may modulate chemotherapy’s antitumor efficacy by influencing PD-L1 levels. Then, we compared the efficacy of DOX between the Cas9 control MC38 group and the CMTM6 KO MC38 group. Comparison of tumor growth curves and final tumor weights revealed that doxorubicin achieved only a 12% tumor suppression rate in the Cas9 control group. However, following PD-L1 KO, the doxorubicin tumor suppression rate increased to 63% (**Figure 5E**). This suggested that tumor PD-L1 expression is also associated with chemotherapy resistance and may represent one of the mechanisms by which CMTM6 regulates the *in vivo* efficacy of chemotherapy.

Taken together, these findings indicated that tumor cell-intrinsic CMTM6 could modulate the *in vivo* efficacy of chemotherapeutic agents, pointing to a potential link between tumor CMTM6 expression and chemoresistance.

Furthermore, our previous studies have demonstrated that CMTM6 is broadly expressed in host cells, especially in immune cells. In 4T1 tumor-bearing mice, doxorubicin treatment markedly upregulated the expression of CMTM6 and PD-L1 in tumor-infiltrating T cells (**Supplementary Figure 4A**). To explore the regulatory function of host cell-derived CMTM6 in chemotherapy efficacy, we established subcutaneous B16F10 tumor models in WT and CMTM6 KO mice and administered doxorubicin intervention. The results revealed that CMTM6 knockout increased the tumor suppression rate of doxorubicin from 38% to 51% (**Supplementary Figure 4B**). Notably, host PD-L1 was depleted, the tumor suppression rate was 57%, and this therapeutic effect was further enhanced to 75% following doxorubicin combination treatment (**Supplementary Figure 4C**). Collectively, both tumor cell- and host cell-expressed CMTM6 and PD-L1 are capable of modulating chemotherapy efficacy.

### The development of adeno-associated virus targeting CMTM6/PD-L1 degradation

The above findings suggested that CMTM6 and PD-L1 may serve as targets for enhancing chemotherapy efficacy. Therefore, first, we planned to develop a gene therapy strategy capable of simultaneously degrading both CMTM6 and PD-L1. We chose a type 9 self-complementary adeno-associated virus (scAAV9) with a short expression time and high infection effectiveness and cloned the particular short hairpin RNA of CMTM6 and PD-L1, known as shCMTM6/PD-L1-scAAV9. To assess the efficacy of shCMTM6/PD-L1-scAAV9 in modulating gene expression in tumor cells, B16F10 cells were infected with this vector at an MOI of 30,000 for a 5-day period. Fluorescence experiments demonstrated that B16F10 cells infected with shCMTM6/PD-L1-scAAV9, which expresses the EGFP fluorescent protein, exhibited green fluorescence, indicating that shCMTM6/PD-L1-scAAV9 can infect tumor cells *in vitro*. Furthermore, flow cytometry analysis revealed a robust suppression of PD-L1 expression in infected cells, with an interference efficiency for CMTM6 reaching 53%. Furthermore, in tumor-bearing mice, shCMTM6/PD-L1-scAAV9 significantly reduced CMTM6 and PD-L1 expression in tumor cells, with interference rates of 20% and 33%, respectively (**Supplementary Figures 5A–D**).

Subsequently, we evaluated the antitumor activity of shCMTM6/PD-L1-scAAV9 in tumor-bearing mice. In the CT26 tumor-bearing mouse model, mice were administered shNC-scAAV9, shCMTM6-scAAV9, shPD-L1-scAAV9, and shCMTM6/PD-L1-scAAV9 via peritumoral injection. Analysis of tumor growth curves and final tumor weights revealed that, compared to the shNC-scAAV9 group, the tumor inhibition rates for the shCMTM6-scAAV9, shPD-L1-scAAV9, and shCMTM6/PD-L1-scAAV9 groups were 50%, 42%, and 76%, respectively (**Supplementary Figures 5E, F**). Subsequent re-evaluation in MC38 and B16F10 tumor-bearing mouse models demonstrated that shCMTM6/PD-L1-scAAV9 also significantly inhibited MC38 tumor growth and prolonged survival in B16F10 tumor-bearing mice (**Figure 6A**; **Supplementary Figures 5H**). These findings collectively indicated that shCMTM6/PD-L1-scAAV9 exerted potent antitumor effects across different murine tumor models.

**Figure 6:**
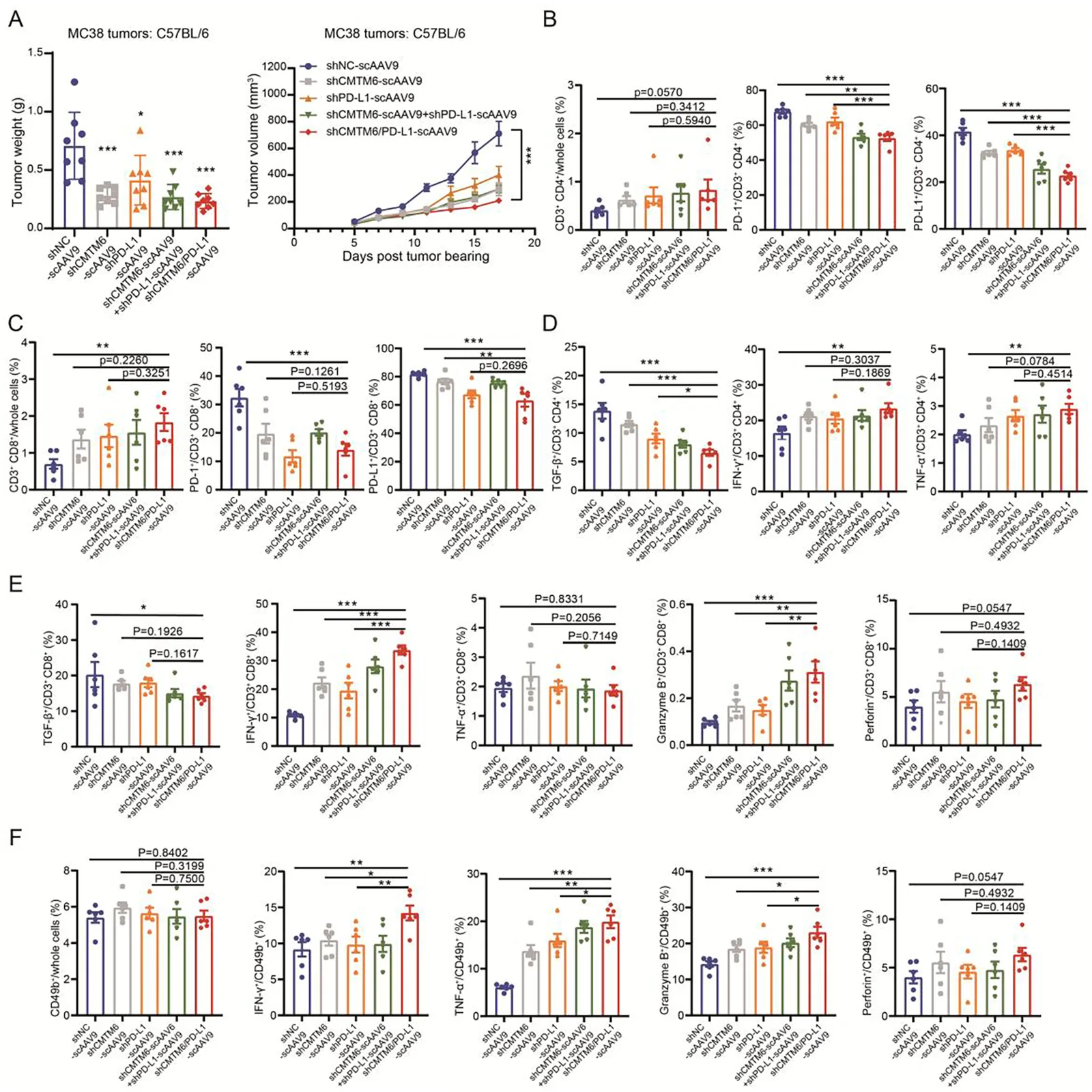
The development of shCMTM6/PD-L1-scAAV9. **(A)** Tumor weight and tumor volume growth curves in MC38 tumor-bearing mice following scAAV9 treatment (n=8). The control group is the shNC-scAAV9 group. **(B–F)** Flow cytometric immunophenotyping analysis of the tumor microenvironment in MC38 tumor-bearing mice following scAAV9 treatment (n=6). The data are presented as the mean ± SEM. *p < 0.05; **p < 0.01; ***p < 0.001 by one-way ANOVA followed by Tukey’s multiple comparisons test.

Then, we performed immune microenvironment flow cytometry analysis for the above CT26 and MC38 tumors. Results showed that in the shCMTM6/PD-L1-scAAV9 treatment group, immune checkpoint molecules such as LAG-3 and PD-1 showed a downregulation trend (**Figures 6B, C**; **Supplementary Figure 5G**), while effector molecules including granzyme B, interferon, and perforin in CD8^+^ T cells and NK cells were significantly upregulated (**Figures 6D–F**; **Supplementary Figure 5G**). Therefore, we concluded that shCMTM6/PD-L1-scAAV9 exhibits potent antitumor activity by improving the tumor immune microenvironment.

### Adeno-associated virus targeting CMTM6/PD-L1 degradation combined with chemotherapy significantly inhibited tumor growth

Subsequently, we evaluated the potential of combining shCMTM6/PD-L1-scAAV9 with chemotherapy drugs. We conducted comparative efficacy studies of the combination therapy in CT26, MC38, and B16F10 tumor-bearing mouse models.

In the B16F10 model, we observed an extension of mouse survival when shCMTM6/PD-L1-scAAV9 was combined with either cisplatin or doxorubicin (**Supplementary Figure 5I**). Notably, the combination of shCMTM6/PD-L1-scAAV9 and doxorubicin resulted in a survival extension of up to 7 days compared to treatment with doxorubicin alone. Compared with the cisplatin monotherapy group, the cisplatin combined with shCMTM6/PD-L1-scAAV9 treatment group extended the survival period of tumor-bearing mice from 21 days to 25 days. In the MC38 model, the combination of shCMTM6/PD-L1-scAAV9 and chemotherapy showed a trend toward tumor suppression compared to the group receiving chemotherapy alone (**Figure 7A**). When treated with shCMTM6/PD-L1-scAAV9 in combination with doxorubicin, the tumor growth inhibition rate reached 85% compared to treatment with shNC-scAAV9 alone, whereas the tumor growth inhibition rate was 79% when used in combination with cisplatin. Similar results were observed in a mouse model bearing CT26 tumors; furthermore, a trend toward tumor suppression was still evident when compared to the group receiving chemotherapy alone (**Figure 7B**; **Supplementary Figure 5J**).

**Figure 7:**
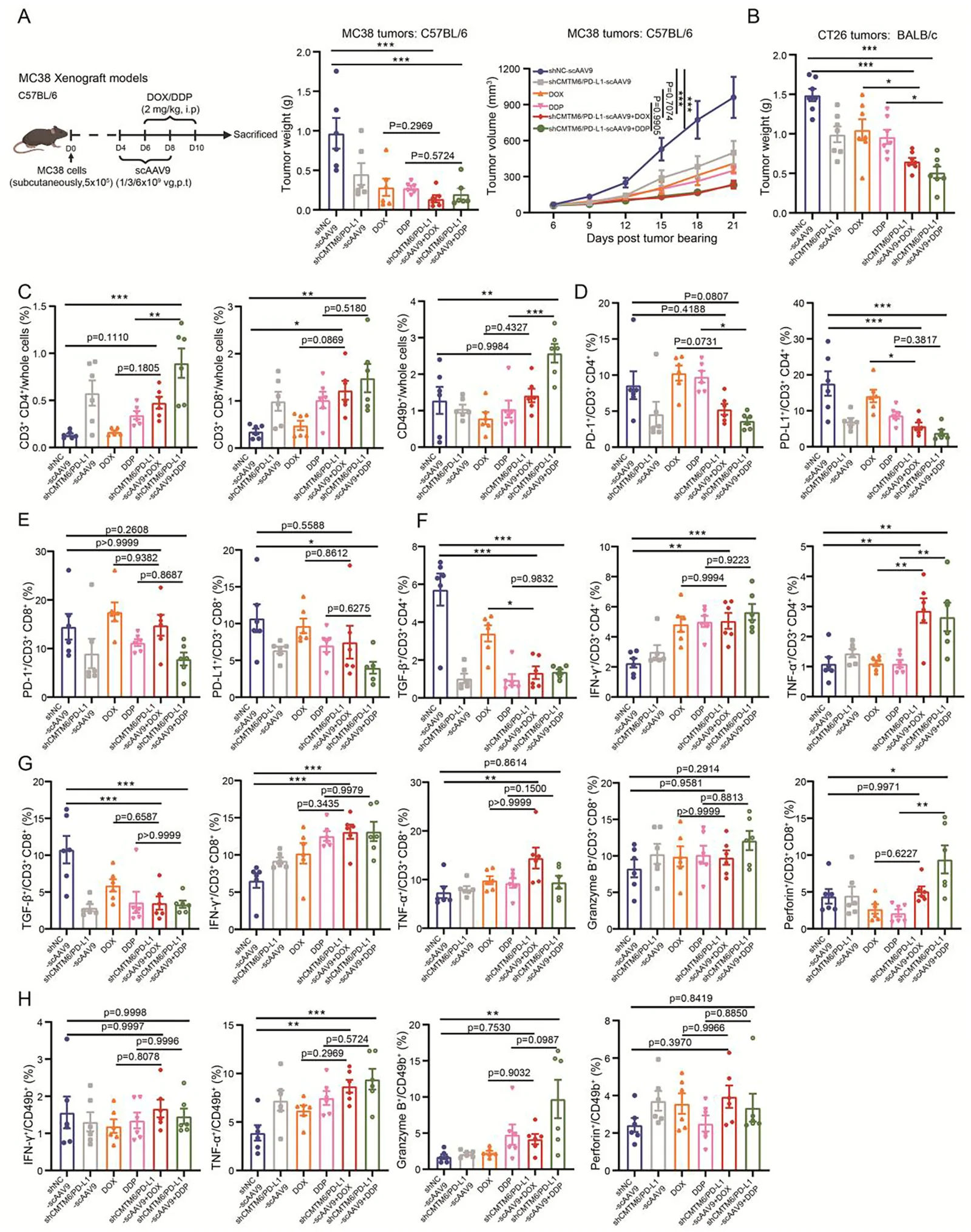
Chemotherapy and shCMTM6/PD-L1-scAAV9 combination therapy significantly inhibited tumor growth. **(A)** Schematic diagram of the combined dosing regimen in MC38 tumor-bearing mice. Tumor growth curves and final tumor weights following combined dosing in MC38 tumor-bearing mice (n=6). **(B)** Final tumor weight in CT26 tumor-bearing mice after combination therapy (n=7). **(C–H)** Flow cytometric immunophenotyping analysis of the tumor microenvironment in MC38 tumor-bearing mice following combined therapy (n=6). The data are presented as the mean ± SEM. *p < 0.05; **p < 0.01; ***p < 0.001 by one-way ANOVA followed by Tukey’s multiple comparisons test.

Then, we performed flow cytometric analysis on subcutaneous tumor tissues isolated from MC38 tumor-bearing mice. Results demonstrated that the combination therapy enhanced the infiltration of CD4^+^ T cells, CD8^+^ T cells, and NK cells into the tumor microenvironment (**Figure 7C**; **Supplementary Figures 6A, B**). Additionally, combination therapy downregulated the expression of inhibitory molecules, including PD-1, PD-L1, and TGF-β, while upregulating the expression of effector molecules such as IFN-γ and TNF-α (**Figures 7D–H**; **Supplementary Figures 6A, B**).

Taken together, shCMTM6/PD-L1-scAAV9 could exert enhanced tumor-suppressive effects when combined with chemotherapy drugs and reshape the tumor immune microenvironment.

## Discussion

In this study, we investigated the effects of chemotherapy on CMMT6 and PD-L1 expression, as well as their impact on chemotherapy resistance. Based on these phenotypic and mechanistic studies, we developed shCMTM6/PD-L1-scAAV9 for combination therapy with chemotherapeutic agents to achieve enhanced antitumor activity.

Chemotherapeutic agents dose- and time-dependently upregulate CMTM6 in tumors, a finding validated in clinical patient samples, *in vitro* human/mouse tumor cells, and *in vivo* mouse tumor models. In our system, tumor PD-L1 expression was also induced by chemotherapy drugs, as reported by other researchers (35, 36). Mechanistically, inhibitor screening preliminarily identified PI3K and mTOR as key mediators of chemotherapy-triggered CMTM6 induction. Existing literature confirms chemotherapeutics activate the mTOR pathway during tumor cell apoptosis: chemotherapy-elicited cell death releases ATP to activate mTOR via P2X4 paracrine signaling (37); chemotherapy-induced DNA damage further stimulates the PI3K/Akt cascade upstream of mTOR. The PI3K-mTOR axis engages mTOR complex 1 (mTORC1) via β-subunit activation, and mTORC1 boosts PD-L1 mRNA translation by activating p70 protein S6 kinase β-1 (p70S6K1) (38, 39). RNA-seq analysis of RKO cells revealed that CMTM6 knockout disrupts multiple signaling pathways associated with drug resistance, including the cell cycle, apoptosis, mTOR, FoxO signaling, and cellular senescence; all of these pathways warrant further investigation. Notably, as previously reported, the mTOR cascade promotes chemotherapy resistance via NF-κB signaling (40). Therefore, we proposed that mTOR and PI3K represented potential pathways through which chemotherapy drugs induced CMTM6 upregulation.

We found CMTM6 knockout downregulates p53 at both transcriptional and protein levels in tumor cells. In previous studies, p53 has been found to activate cytoprotective programs that produce resistance to chemotherapy and radiotherapy. Upon chemotherapy-triggered DNA damage, activated p53 upregulates p21 to arrest the cell cycle, allowing tumor cells to repair chemotherapy-induced DNA double-strand breaks and survive drug treatment. Meanwhile, p53 transcriptionally activates multiple repair genes, including DDB2, BRCA, and MLH1, boosting base excision and homologous recombination repair to counteract chemotherapy-mediated DNA injury. It additionally drives glutathione and antioxidant protein production to neutralize chemotherapy-derived ROS and blunt drug cytotoxicity. Moreover, p53 induces circASCC3 expression to further enhance tumor resistance to chemotherapy (41–43). Therefore, we speculated that tumor CMTM6 may also mediate chemotherapy resistance by regulating p53 levels.

It is worth noting that p53 has also been identified as a canonical tumor suppressor that inhibits tumor development in multiple ways. Classically, when stress signals occur—such as oncogene activation or DNA damage—p53 is activated. The p53 protein mediates apoptosis by transcriptionally activating target genes (such as p21, which is involved in permanent or transient cell cycle arrest) and pro-apoptotic members of the BCL2 family (such as Noxa and BBC3) (44). In addition, p53 exerts its antitumor function by inhibiting glycolysis in tumor cells (45). Other studies have reported that p53 can also induce the expression of miR-200c and miR-34a to block epithelial-mesenchymal transition (EMT) and thereby inhibit tumor progression (46, 47). However, studies have shown that low doses of doxorubicin cause only sublethal DNA damage; although this induces p53 accumulation, p53 at this stage is primarily involved in DNA damage repair and cell cycle arrest, which promotes cell survival. Only when doxorubicin reaches a lethal dose and induces extensive, irreparable DNA damage does p53 effectively promote apoptosis (48). Therefore, we hypothesize that this may be a potential explanation for the aforementioned conflicting functions.

We considered that chemotherapy can upregulate CMTM6 and PD-L1 in tumor cells and that the absence of these two molecules can counteract their immunosuppressive signals. In CMTM6-knockout 4T1 tumors, doxorubicin markedly elevates antitumor TNF-α in tumor-infiltrating T cells, validating this hypothesis. These observations agree with our prior report establishing CMTM6 as a novel tumor immune regulatory molecule (30). We additionally confirmed host loss of CMTM6 and PD-L1 improves chemotherapeutic outcomes. Chemotherapeutics upregulate CMTM6 and PD-L1 across host tissues, whereas host deficiency of these molecules reverses their immunosuppressive effects. However, further research is needed to explore other potential mechanisms.

In selecting a tumor treatment strategy, we opted for the scAAV formulation. This approach can simultaneously reduce the expression of both CMTM6 and PD-L1. Although chemotherapy upregulates the co-expression levels of CMTM6 and PD-L1 in tumors by less than 30%, our previous research found that the absence of CMTM6 reduces tumor PD-L1 membrane expression by only 50%, while also inducing a PD-L1-independent antitumor immune response (29). Based on these considerations, we selected shCMTM6/PD-L1-scAAV9 in combination with chemotherapy as the treatment strategy. In the *in vivo* efficacy assessments, shCMTM6/PD-L1-scAAV9 indeed demonstrated stronger antitumor activity than the use of CMTM6 or PD-L1 interference alone. Furthermore, shCMTM6/PD-L1-scAAV9 and chemotherapy combination demonstrated significant antitumor effects. These effects were primarily achieved through the following three mechanisms: upregulation of the infiltration of CD4^+^ T cells, CD8^+^ T cells, and NK cells; downregulation of the expression of inhibitory molecules such as PD-1, PD-L1, and TGF-β on T cells; and enhancement of the expression of effector molecules on CD8^+^ T cells and NK cells, thereby improving the antitumor activity of the immune microenvironment.

Of course, our study has many limitations. First, we did not elucidate the specific regulatory pathway underlying chemotherapy drug-induced CMTM6 expression in tumor cells. Although we hypothesize that PI3K and mTOR play a role in this process, systematic validation remains necessary in subsequent studies. Second, we have not delineated the mechanism whereby CMTM6 knockout reverses chemoresistance in tumor cells. While we show that CMTM6 depletion modulates PD-L1 expression, we lack formal evidence that CMTM6 specifically regulates chemotherapy sensitivity via PD-L1. We plan to perform PD-L1 rescue experiments in future work to distinguish PD-L1-dependent and PD-L1-independent mechanisms underlying CMTM6-mediated chemoresistance. Likewise, we have not confirmed whether the p53 pathway contributes to this phenotypic reversal, which can be interrogated via p53 rescue assays in follow-up studies. Previous reports have demonstrated that CMTM6 promotes chemoresistance through Wnt and Myc signaling pathways (49, 50). In future studies, we will systematically investigate the molecular mechanisms by which CMTM6 mediates chemotherapy resistance, taking into account relevant signaling pathways. Third, our experiments revealed that host CMTM6 and PD-L1 continued to exert significant regulatory effects on chemotherapy efficacy. Whether the antitumor activity of host CMTM6 during chemotherapy depends on the regulatory mechanism of host PD-L1 remains to be explored in subsequent studies. Comparing the efficacy differences of chemotherapy drugs in PD-L1 KO mice and PD-L1&CMTM6 KO mice may help explain this issue. Furthermore, there are still many limitations to the use of scAAV as a delivery system for cancer therapy. For example, the double-stranded genomic structure of scAAV results in a packaging capacity of only about 2.2 kb, which severely limits the options for vector construction (51); upon systemic administration, scAAV still preferentially accumulates in the liver, has limited efficiency in crossing the blood-brain barrier, and is prone to inducing liver damage and activation of the innate immune system at high doses (52, 53); The widespread presence of AAV neutralizing antibodies in the population can completely block vector delivery (54); AAV preparation is subject to genomic heterogeneity, and the packaging process is prone to generating large amounts of single-stranded impure genomes, which reduces the proportion of functional vectors and further lowers the efficiency of effective *in vivo* delivery (55). Therefore, the shCMTM6/PD-L1-scAAV9 treatment regimen still requires further evaluation in terms of dosage, safety, and the diversity of tumor models, and a comparison of its efficacy with that of clinically used anti-PD-1/PD-L1 antibodies will need to be conducted.

In summary, chemotherapy drugs could induce the expression of CMTM6 in tumor cells, with mTOR and PI3K serving as potential regulatory pathways. The downregulation of p53 caused by CMTM6 deficiency may represent a potential mechanism for overcoming chemotherapy resistance. Tumor or host CMTM6 could modulate chemotherapy-induced PD-L1 expression, tumor cell apoptosis, tumor cell growth inhibition, and the efficacy of chemotherapeutic agents. Moreover, we developed shCMTM6/PDL1-scAAV9 to interfere with tumor CMTM6 and PD-L1 expression. Administering shCMTM6/PD-L1-scAAV9 in combination with chemotherapy across multiple tumor models significantly enhances antitumor effects by improving the tumor microenvironment. Our research suggests that CMTM6 and PD-L1 may serve as therapeutic targets for improving chemotherapy resistance or chemotherapy combination strategy, demonstrating significant clinical application potential.

## Data Availability

The data presented in the study are deposited in the NCBI BioProject repository, accession number PRJNA1525092.
