# Supplementary material for: CMTM6/PD-L1-targeted AAV promotes anti-tumor immune responses and chemotherapy responsiveness
Source: Front Immunol. 2026 Sep 14;17:1886034. doi: 10.3389/fimmu.2026.1886034 (PMC13617519; doi:10.3389/fimmu.2026.1886034)
Supplement: Supplementary file 1 [file DataSheet1.pdf]

Supplementary Materials

Title: CMTM6/PD-L1-Targeted AAV Promotes Anti-tumor Immune Responses and Chemotherapy Responsiveness

Authors: Runqiu Chen[1], Xiaoyu Pang[2], Fanglin Li[3], Shanna Wu[4], Yiting Wang[3], Xinhang Zheng[3,5], Yaqi Zhao[2,3], Fengru Qiu[3], Liying Chen[3], Fengyu Du[3,5], Chen Chen[3,5] Rong Zhang[3], Zhiying Li[3], Yiru Long[3 *], Likun Gong[2,3,4,6 *], Meiqing Feng[1,7 *]

This file contains:

Table S1 Antibody reagents

Figure S1 Analysis of clinical tumor response to chemotherapy based on CMTM6 and PD-L1 expression.

Figure S2 Relationship between CMTM6 and PD-L1 expression in tumor cells following co-incubation with chemotherapy drugs.

Figure S3 Chemotherapy drugs induced tumor CMTM6 and PD-L1 expression and potential regulatory mechanisms.

Figure S4 Host CMTM6 or PD-L1 deficiency enhanced doxorubicin efficacy.

Figure S5 shCMTM6/PD-L1-scAAV9 interfered with CMTM6 and PD-L1 expression and inhibited tumor growth.

Figure S6 Flow cytometry gating strategies for analyzing the tumor microenvironment following combination therapy with chemotherapy and shCMTM6/PD-L1-scAAV9.

Supplementary table

Table S1. Antibody reagents

**Reagents**	**Source**	**Identifier**
FITC Rat Anti-Mouse CD45	BD Pharmingen	560694
FITC Rat anti-Mouse CD3	BD Pharmingen	553062
APC-Cy7 Rat anti-Mouse CD3	BD Pharmingen	557596
BV421 Rat Anti-Mouse CD4	BD Pharmingen	562891
BV510 Hamster Anti-Mouse CD8a	BD Pharmingen	563068
BV650 Rat Anti-Mouse CD274	BD Pharmingen	740614
BV786 Rat Anti-Mouse CD274	BD Pharmingen	741014
BV786 Rat Anti-Mouse CD279	BD Pharmingen	744548
FITC Rat Anti-Mouse CD49b	BD Pharmingen	553857
BV786 Rat Anti-Mouse IFN-γ	BD Pharmingen	563773
BV650 Rat Anti-Mouse TNF	BD Pharmingen	563943
PE Rat Anti-Mouse LAG3	BD Pharmingen	552380
PE anti-mouse LAP (TGF-β1)	BioLegend	106323
p53 (1C12) Mouse Monoclonal Antibody (Alexa Fluor ® 647 Conjugate)	CST	2533S
ANTI-M GRANZYME B NGZB PE-CYN7	Thermo	25-8898-82
ANTI-MO PERFORIN EBIOOMAKD APC	Thermo	17-9392-80
Anti-CMTM6 antibody	Abcam	ab264067
Goat Anti-Rabbit IgG H&L (Alexa Fluor® 647)	Abcam	ab150079

Supplementary figures

Figure S1

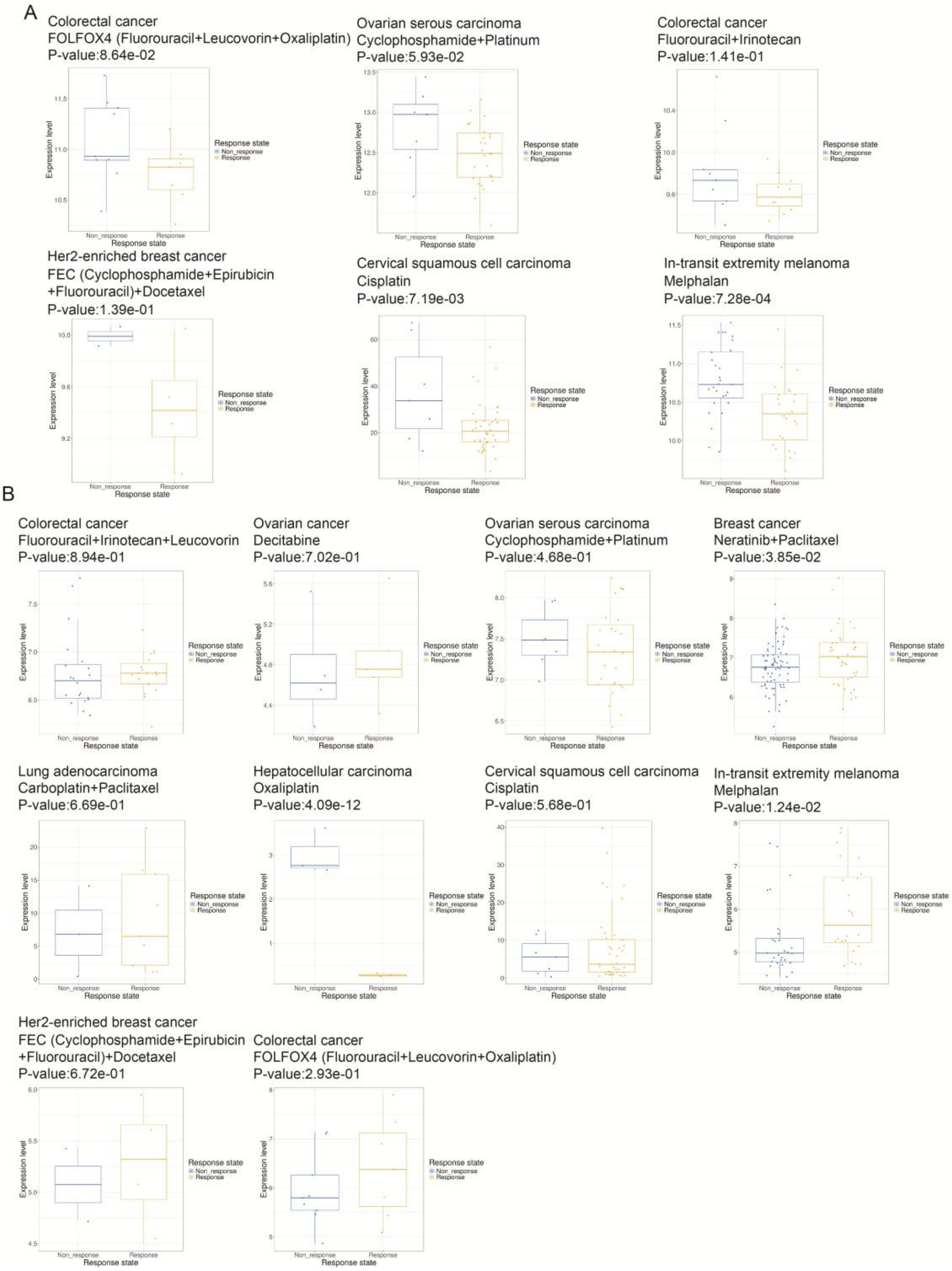

Fig S1. Analysis of clinical tumor response to chemotherapy based on CMTM6 and PD-L1 expression. (A) Correlation between tumor CMTM6 expression and chemotherapy response in multiple tumor types. (B) Correlation between tumor PD-L1 expression and chemotherapy response in multiple tumor types. The data are presented as the mean ± SEM by unpaired t test.

Figure S2

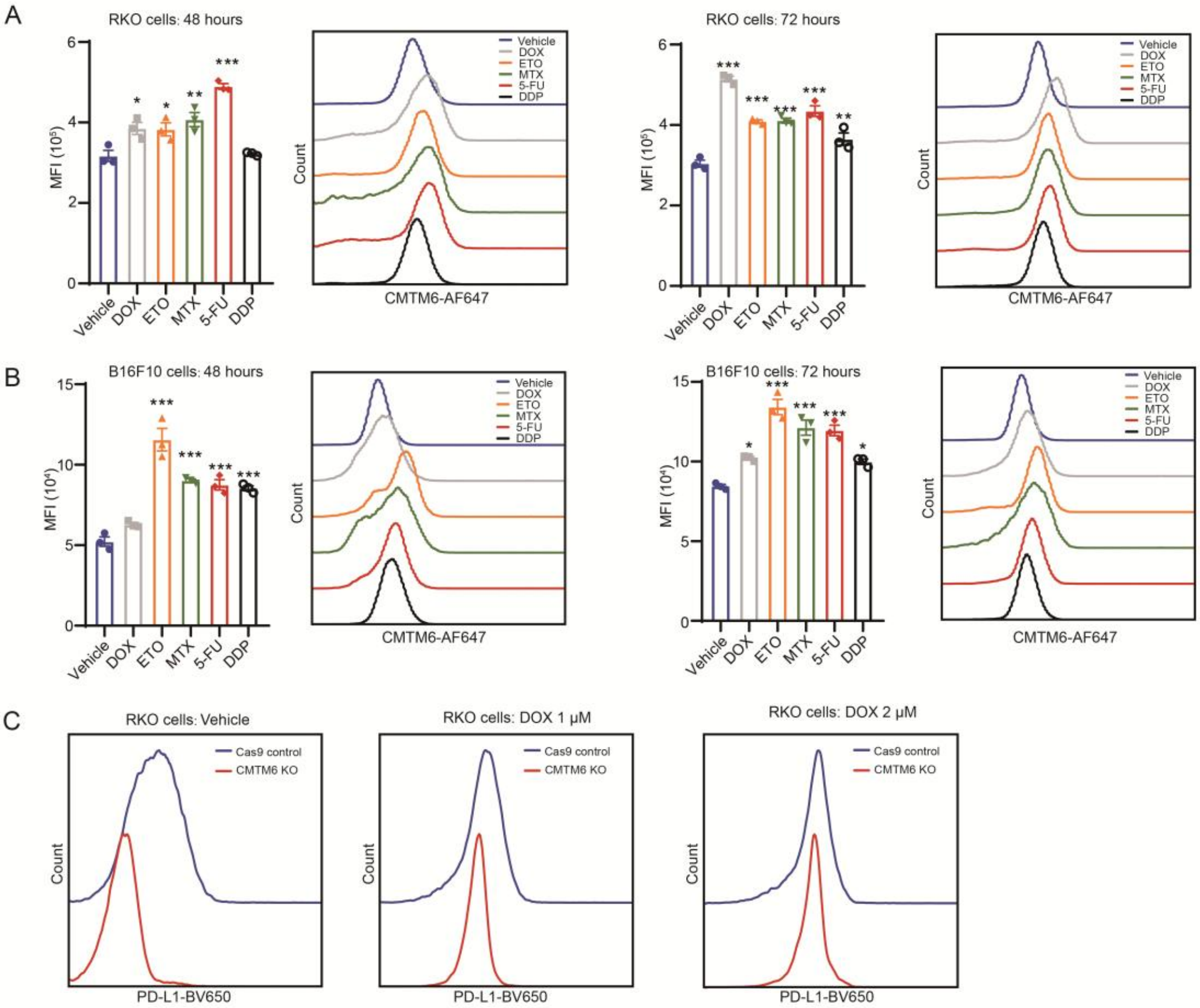

Fig S2. Relationship between CMTM6 and PD-L1 expression in tumor cells following co-incubation with chemotherapy drugs. (A, B) CMTM6 expression and representative flow cytometry plots in RKO and B16F10 cell models after 48-hour and 72-hour incubation with chemotherapeutic agents (A, B: n=3). (C) Representative flow cytometry plots of PD-L1 expression at different incubation doses of doxorubicin. The data are presented as the mean ± SEM. * $p < 0.05$; ** $p < 0.01$; *** $p < 0.001$; ns not significant by one-way ANOVA followed by Tukey's multiple comparisons test.

Figure S3

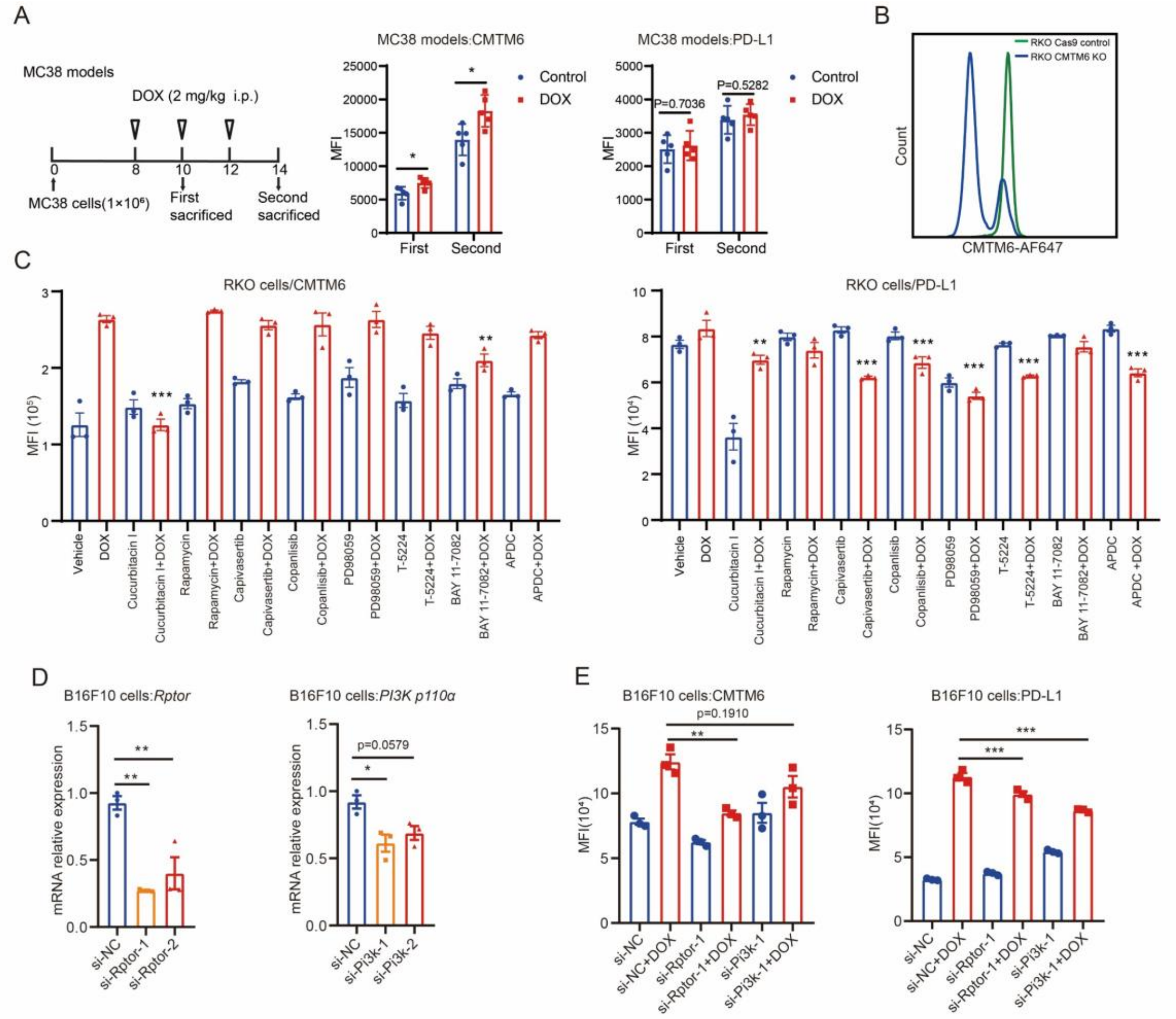

Fig S3. Chemotherapy drugs induce tumor CMTM6 and PD-L1 expression and potential regulatory mechanisms. (A) CMTM6 and PD-L1 expression in tumors of MC38 tumor-bearing mice during doxorubicin treatment (n=5). (B) Identification of RKO cell lines with Cas9 Control and CMTM6 knockout. (C) Expression of CMTM6 and PD-L1 in RKO cells after 48 h treatment with various pathway inhibitors and DOX (n=3). The control group is the DOX group. (D) Expression of Rptor and PI3K p110α in B16F10 cells following siRNA knockdown (n=3). (E) Expression of CMTM6 and PD-L1 in B16F10 cells following siRNA knockdown and DOX treatment (n=3). The data are presented as the mean ± SEM. * $p < 0.05$; ** $p < 0.01$; *** $p < 0.001$; ns not significant by one-way/two-way ANOVA followed by Tukey’s multiple comparisons test or multiple t tests.

Figure S4

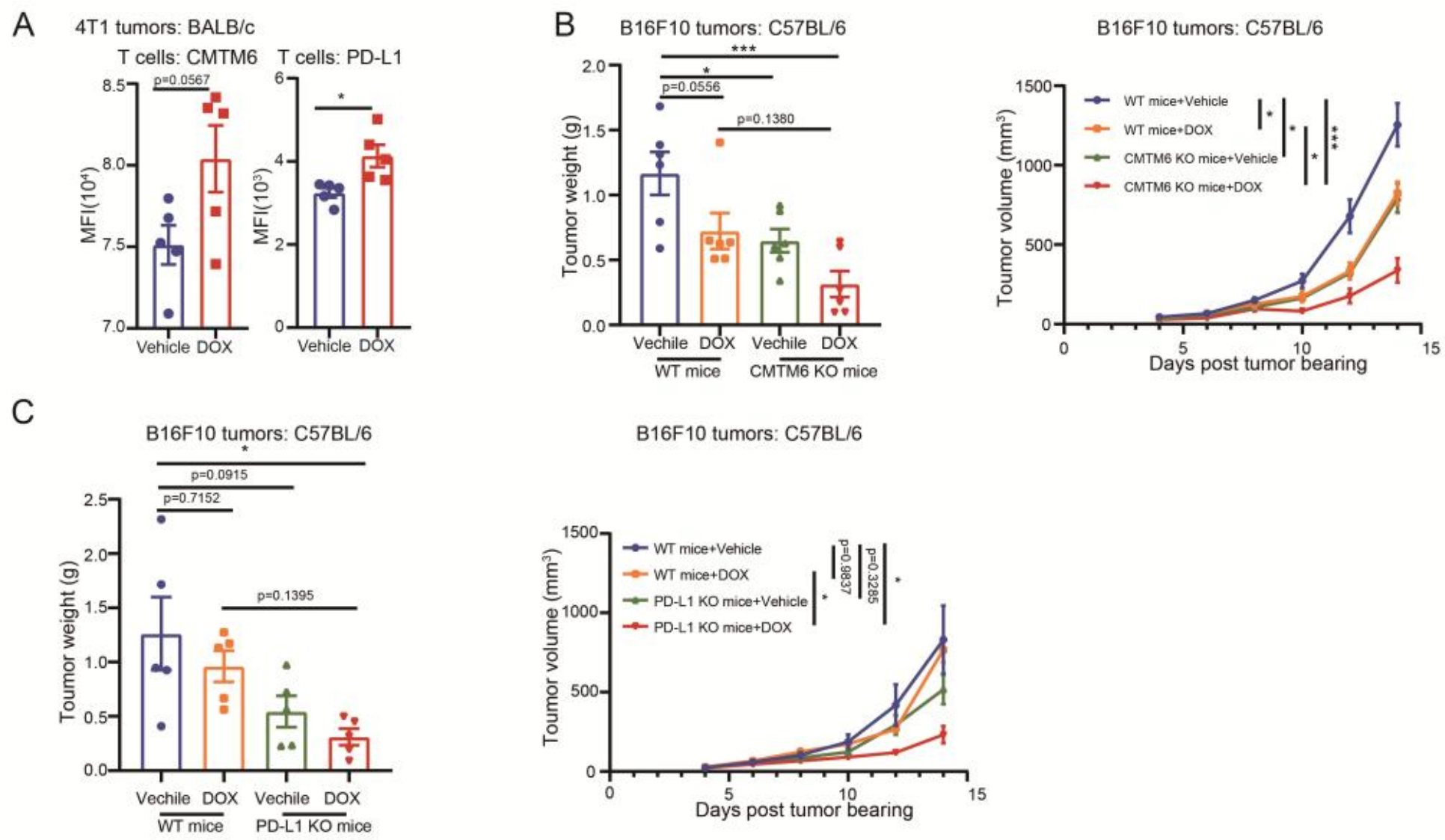

Fig S4. Host CMTM6 or PD-L1 deficiency enhanced doxorubicin efficacy. (A) Expression of CMTM6 and PD-L1 on T cells in the immune microenvironment of 4T1 tumor-bearing mice treated with doxorubicin (n=5). (B) End-point tumor weight and tumor growth curves following doxorubicin treatment in WT and CMTM6 KO C57BL/6 mice bearing B16F10 tumors (n=7). (C) End-point tumor weight and tumor growth curves following doxorubicin treatment in WT and PD-L1 KO C57BL/6 mice bearing B16F10 tumors (n=5). The data are presented as the mean ± SEM. * $p < 0.05$; *** $p < 0.001$ by unpaired t test or one-way/two-way ANOVA followed by Tukey's multiple comparisons test.

Figure S5

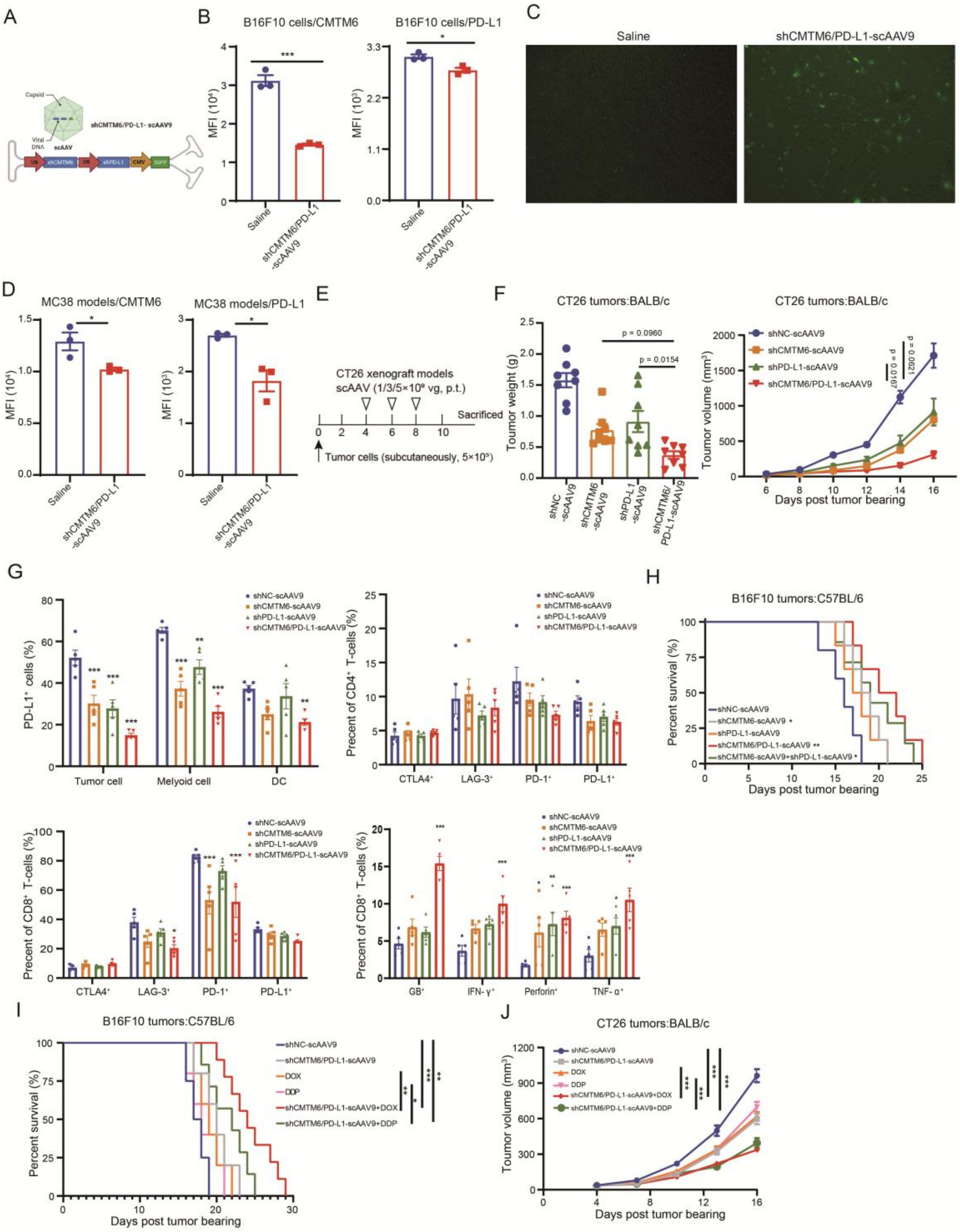

Fig S5. shCMTM6/PD-L1-scAAV9 interferes with CMTM6 and PD-L1 expression and inhibits tumor growth. (A) Schematic diagram of shCMTM6/PD-L1-scAAV9 construction. (B) CMTM6 and PD-L1 expression in B16F10 cells following shCMTM6/PD-L1-scAAV9 treatment (n=3). (C) EGFP expression in B16F10 cells following shCMTM6/PD-L1-scAAV9 treatment. (D) Expression of CMTM6 and PD-L1 in tumor tissues of MC38 tumor-bearing mice following treatment with shCMTM6/PD-L1-scAAV9 (n=3). (E) Schematic diagram of the CT26 tumor-bearing mouse scAAV administration protocol. (F) Tumor growth curves and final tumor weights in CT26 tumor-bearing mice following scAAV treatment (n=8). (G) Flow Cytometric

Immunophenotyping Analysis of the Tumor Microenvironment in CT26 Tumor Mice Following scAAV Treatment (n=5). The control group is the shNC-scAAV9 group. (H) Survival curve of B16F10 tumor-bearing mice after scAAV treatment (n=10). The control group is the shNC-scAAV9 group. (I) Survival curve of B16F10 tumor-bearing mice following combined therapy (n=10). The control group is the shNC-scAAV9 group. (J) Tumor growth curve of CT26 tumor-bearing mice after combination therapy (n=7). The data are presented as the mean ± SEM. * $p < 0.05$; *** $p < 0.001$ by unpaired t test or one-way/two-way ANOVA followed by Tukey's multiple comparisons test. Survival curves were estimated by the Kaplan–Meier method. Overall intergroup differences were assessed using the Log-rank test. * $p < 0.05$; ** $p < 0.01$; *** $p < 0.001$.

Figure S6

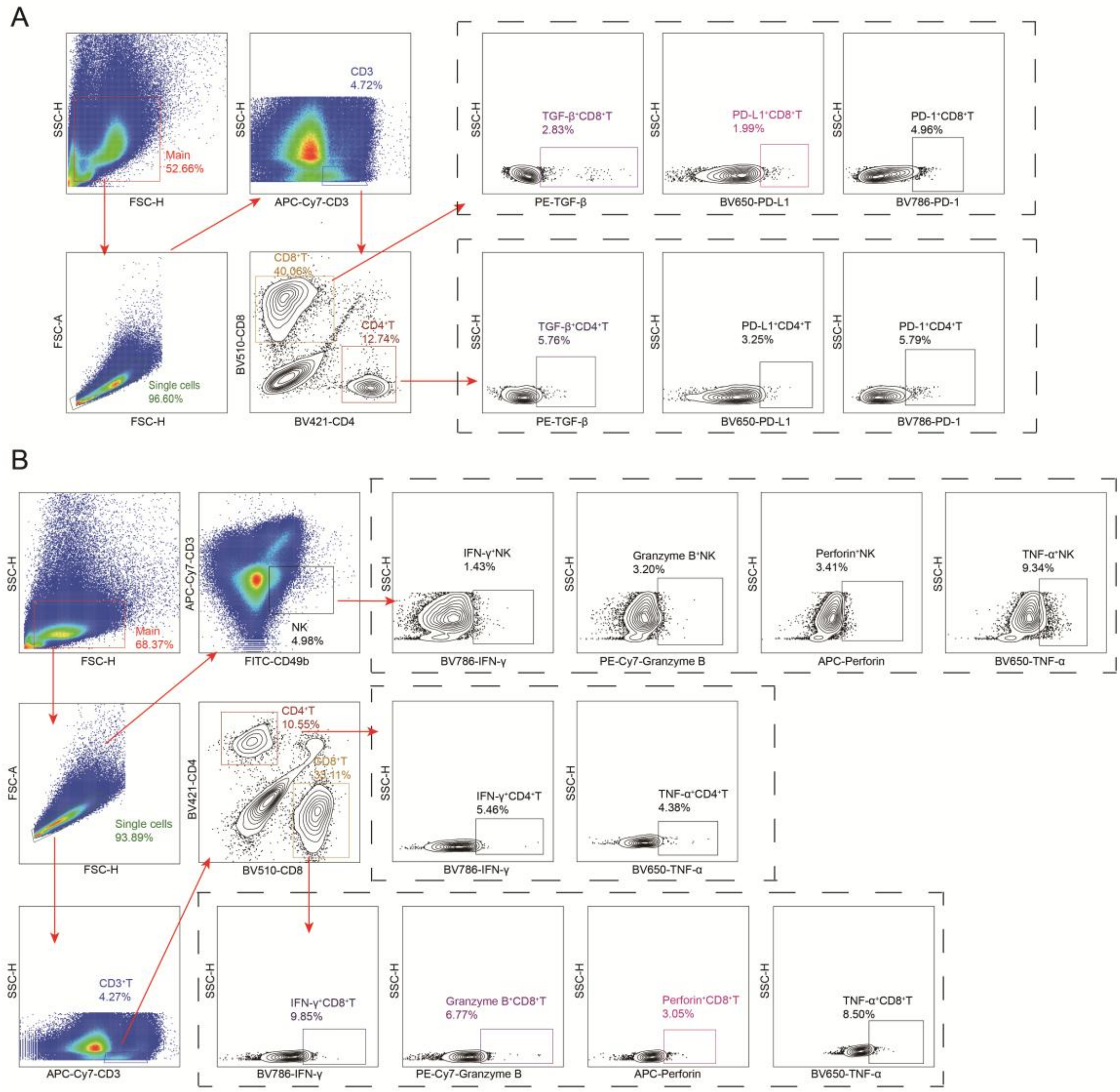

Fig S6. Flow cytometry gating strategies for analyzing the tumor microenvironment following combination therapy with chemotherapy and shCMTM6/PD-L1-scAAV9. (A-B) Flow cytometric immunophenotyping strategy for the tumor microenvironment in MC38 tumor-bearing mice following combination therapy.
